# A genetically encoded fluorescent heme sensor detects free heme in plants

**DOI:** 10.1093/plphys/kiae291

**Published:** 2024-05-19

**Authors:** Bingxiao Wen, Bernhard Grimm

**Affiliations:** Institute of Biology/Plant Physiology, Humboldt-Universität zu Berlin, 10115 Berlin, Germany; Institute of Biology/Plant Physiology, Humboldt-Universität zu Berlin, 10115 Berlin, Germany

## Abstract

Heme is produced in plants via a plastid-localized metabolic pathway and is subsequently distributed to all cellular compartments. In addition to covalently and noncovalently bound heme, a comparatively small amount of free heme that is not associated with protein is available for incorporation into heme-dependent proteins in all subcellular compartments and for regulatory purposes. This “labile” fraction may also be toxic. To date, the distribution of the free heme pool in plant cells remains poorly understood. Several fluorescence-based methods for the quantification of intracellular free heme have been described. For this study, we used the previously described genetically encoded heme sensor 1 (HS1) to measure the relative amounts of heme in different plant subcellular compartments. In a proof of concept, we manipulated heme content using a range of biochemical and genetic approaches and verified the utility of HS1 in different cellular compartments of *Arabidopsis* (*Arabidopsis thaliana*) and tobacco (*Nicotiana tabacum* and *Nicotiana benthamiana*) plants transformed either transiently or stably with HS1 and HS1(M7A), a variant with lower affinity for heme. This approach makes it possible to trace the distribution and dynamics of free heme and provides relevant information about its mobilization. The application of these heme sensors will create opportunities to explore and validate the importance of free heme in plant cells and to identify mutants that alter the subcellular allocation of free heme.

## Teaser

A fluorescent heme sensor predicts the level of free heme in different subcellular compartments of *Arabidopsis thaliana*, *Nicotiana tabacum*, and *Nicotiana benthamiana*.

## Introduction

Heme is an essential cofactor for many metabolic functions in all subcellular compartments. It is used for redox reactions, binding of gases, and signaling events. Heme is synthesized in the tetrapyrrole biosynthesis (TBS) pathway, which in plants is exclusively localized in plastids (Tanaka and Tanaka 2007; Terry and Smith 2013; Hu et al. 2021; Wang et al. 2022). Apart from heme, TBS in plants is responsible for the formation of chlorophyll, siroheme, and phytochromobilin in widely varying quantities in the different plant organs. Following the synthesis of heme in plastids, it must be transferred across the 2 plastidal envelope membranes, and after its release from plastids, heme needs to be distributed to the different cellular compartments.

Heme that is bound to high-affinity heme-dependent proteins represents the dominant fraction of this metabolite in all cells. This bound fraction is essentially employed for cellular functions and is not readily exchangeable between heme-binding proteins to support different heme-dependent functions. On the other hand, levels of “labile” and free heme must be tightly controlled owing to the toxicity of the latter. Nevertheless, free heme must remain available for different functions, so that it can be mobilized for trafficking between and signaling within subcellular compartments.

Several methods have been proposed for the assessment of the level of either total heme or free heme pools in plant tissue, and it is known that only noncovalently bound and the relatively small amount of free heme molecules are accessible to quantification in cells (Masuda and Takahashi 2006; Espinas et al. 2012; Richter et al. 2019). In one of the available approaches, heme content is estimated by determining the amount of the compound released from chloroplasts by apo-horseradish peroxidase (Weinstein and Beale 1983). However, the quantitative determination of heme content in plants is difficult, not only because of the large numbers of tetrapyrrole end products involved. Rapid and reliable ways to quantify the total steady-state heme content of a tissue or even a specific class of subcellular compartment are lacking—quite apart from the problems posed by the quantitative differentiation between “kinetically inert” (i.e. bound) heme and free/labile heme. Most importantly, no method is available for the quantification of differences between heme contents at the subcellular level in plants. Only the amount of noncovalently bound heme (mainly b-type heme) relative to the total amount of heme can be ascertained in plant cells—following homogenization and extraction from the tissue. Thus, no rapid purification methods are available for the subcellular determination of heme extracted from purified organelles.

The steady-state level of free heme is expected to be tightly controlled, so as to avoid its pro-oxidative and cytotoxic effects. This applies not only to heme synthesis but also to the mobilization and transport of heme and its subcellular distribution, which ensure the availability of free heme for incorporation into heme-dependent proteins. Nothing is known about the mechanisms or the factors involved in the control of heme distribution within plant cells nor how newly synthesized heme derived from the 2 ferrochelatase isoforms in plastids is assembled into heme-dependent proteins.

Considering the numerous open questions regarding the metabolism of heme and its subcellular allocation in plants, even methods for the determination of the amounts of free heme in the subcellular compartments of plant cells would be of great value. Control of the bioavailability of labile heme, including rates of translocation across membranes for further subcellular distribution, determines heme-dependent signaling and the catalytic functions of heme-dependent enzymes. Similarly, not much is known about the regulation of heme synthesis and posttranslational control of heme accumulation during the synthesis of the 2 ferrochelatase isoforms or the control of heme degradation in plants. It is thought that heme concentration is primarily determined by the relative rates of heme synthesis. But how feedback control of heme synthesis operates to determine the appropriate bioavailability of free heme amounts in all cellular compartments is completely open.

A sensitive and effective method for measuring bioavailable free heme pools in different subcellular compartments was presented using a genetically engineered fluorescent heme sensor 1, named and abbreviated HS1 (Hanna et al. 2016). HS1 is a tripartite fusion protein consisting of cytochrome b_562_ (Cytb_562_) from *Escherichia coli* and the enhanced GFP (EGFP) and the far-red fluorescent protein mKATE2 (Katushka 2, hereinafter called mKATE2). When the Cytb_562_ moiety of the sensor binds to heme, it serves as an acceptor for resonance energy transfer from EGFP, while mKATE2 fluorescence is insensitive to heme (Hanna et al. 2016). The response to noncovalent binding of heme to Cytb_562_ is therefore based on the fluorescence quenching that occurs when heme binds to HS1. The ratio between the heme-sensitive EGFP fluorescence and the stable, heme-insensitive mKATE2 fluorescence thus varies depending on the amount of free heme available. Therefore, HS1 enables the ratiometric imaging of heme in intact cells and their subcellular compartments and circumvents the need for cell disruption and time-consuming enzyme assays (Hanna et al. 2016). Moreover, since it is expected that different amounts of heme are found in different organelles, Hanna and coworkers designed a variant of the HS1 sensor with a lower affinity for heme in order to more reliably assess the relative contents of free heme in different subcellular compartments. Thus, the system provides a heme-specific tool that enables the spatial and temporal visualization of the intracellular distribution of free heme to be quantified and allows one to predict the dynamics and follow the mobilization and trafficking of heme (Hanna et al. 2016).

We set out to adapt the heme sensor HS1 for use in plants, which contain with plastids an additional organelle that is not found in either yeast or animal cells, and to assess the relative amounts of free heme in different subcellular compartments. We began by designing and subcloning various gene constructs that enabled the heme sensor to be translocated into the various subcellular compartments. Several lines of evidence are presented that illustrate the ability of these HS1 constructs to quantify the relative amounts of freely accessible intracellular heme and detect dynamic changes in the subcellular heme content in plants that have been exposed to various genetic, biochemical, and environmental perturbations.

## Results

**Figure 1.**
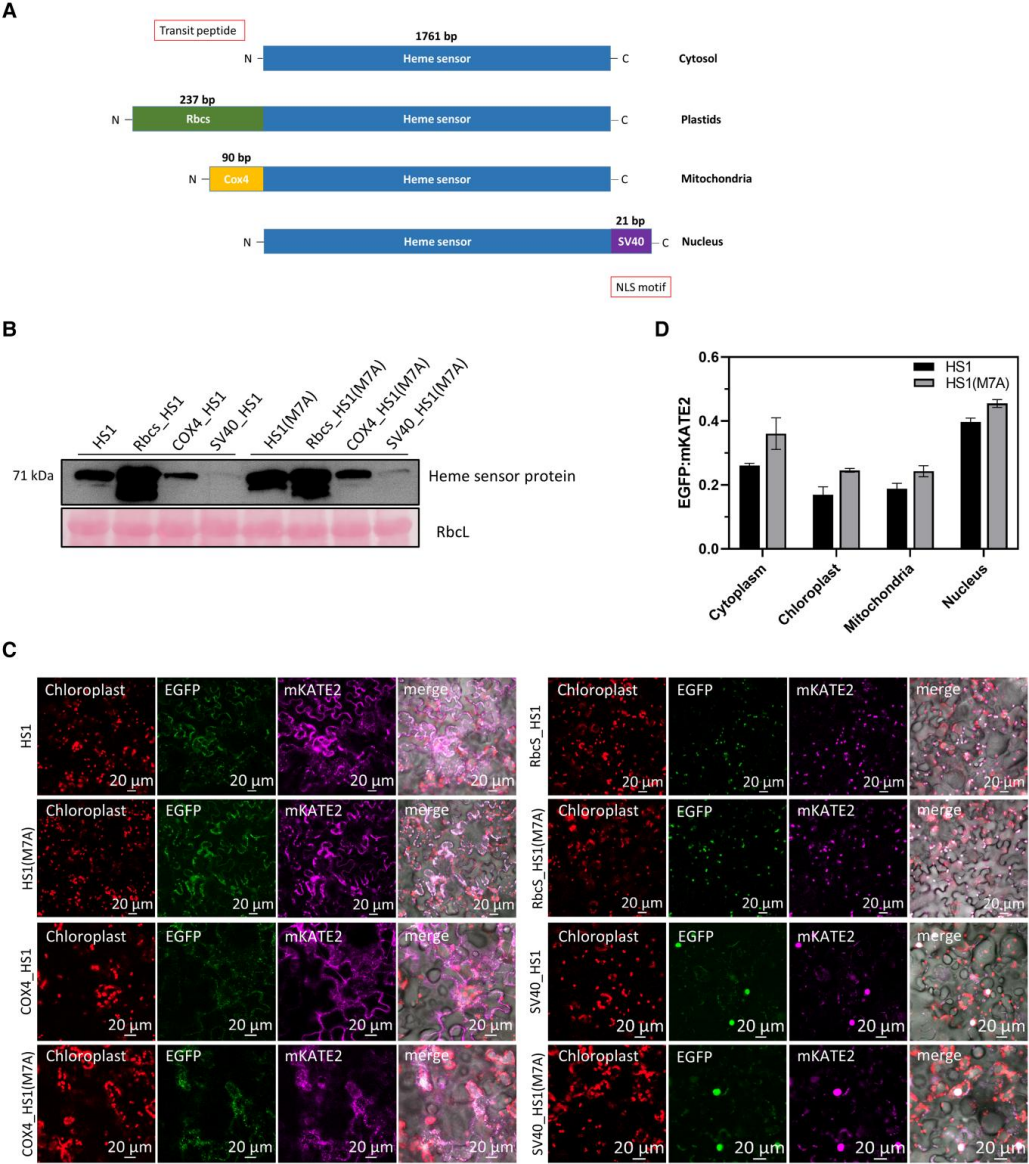
Quantification of the relative amounts of the 2 heme sensors, both of which incorporate the fluorescent proteins EGFP and mKATE2 as reporter and internal standard, respectively. The 2 heme sensor variants HS1 and HS1(M7A), which differ in their respective affinities for heme, were transiently expressed in different subcellular compartments of *N. benthamiana*. **A)** The coding sequences of the 2 heme sensors themselves facilitate their expression in the cytoplasm. Sequences coding for the transit peptides of RbcS and COX4 were added to the sensors to enable them to be targeted to plastids and mitochondria, respectively, and transportation into the nucleus was assured via the nuclear localization signals of SV40. The exact length of the coding sequences for the heme sensor and the respective transit peptides and the signal sequence is given in base pairs. **B)** Immunoblot analysis of the 2 heme sensor variants, which were targeted to different cellular compartments. Samples of protein extracts from transiently transformed *N. benthamiana* leaves were analyzed by using the anti-GFP antibody, and the Coomassie-stained large subunit of RbcL served as a loading standard. **C)** The fluorescent signals emitted by HS1 (the more sensitive heme sensor) and HS1(M7A) (which has a lower affinity for free heme) in different subcellular compartments in transiently transformed *N*. *benthamiana* leaves. Apart from chlorophyll fluorescence, the images show representative EGFP and mKATE2 signals and their merger recorded by the HS1 variants in the 4 cellular compartments. For transient transformation, 6-wk-old plants were used, and the leaves used for transfection were all of the same age. **D)** The ratio of EGFP to mKATE2 fluorescence (EGFP/mKATE2) recorded by HS1(M7A) and HS1 is shown for the different cellular compartments. The EGFP/mKATE2 ratios obtained from the EGFP and mKATE2 channels were calculated from 5 to 8 transformed representative cells of different transformants. Note that the ratio is inversely proportional to the level of free heme (see main text). Statistical significance compared with fluorescence in the cytoplasm is indicated by Tukey's HSD method (*P < 0.05, **P < 0.01), and error bars represent the sd of 3 biological replicates.

Gene constructs were designed for the expression and translocation of the heme sensor variants HS1 and HS1(M7A), which are characterized by different heme-binding affinities (Hanna et al. 2016), and were used to assay the levels of free heme in 4 different subcellular compartments of plants (cytoplasm, plastids, mitochondria, and nucleus). Translocation of the heme sensor variants into plastids, mitochondria, and nucleus was achieved by using the coding sequences for the transit peptides of small subunit of ribulose bisphosphate carboxylase (RbcS) and cytochrome c oxidase subunit 4 (COX4) and the nuclear translocation signal peptide of the DNA virus SV40, respectively. The relative amounts of HS1 and HS1(M7A) in each compartment approximately correspond to the volume of each compartment (Fig. 1). Because the affinity of HS1(M7A) for heme is lower than that of HS1, the ratio of EGFP to mKATE2 fluorescence in all cellular compartments was always higher than that of HS1-expressing plants. This confirms the applicability and utility of the transiently expressed heme sensor variants for the assessment of the relative amounts of freely available heme in plant organelles. Moreover, the system will also allow us to monitor heme levels in mutants with altered heme metabolism and their effects on heme trafficking.

The differences in the ratios of EGFP to mKATE2 fluorescence reported by HS1 relative to HS1(M7A) were fairly similar in all subcellular compartments (Fig. 1C), except the nucleus, in which this parameter was systematically lower. As the EGFP/mKATE2 fluorescence ratios in nuclei were always the highest, we suggest that this is most likely due to a very low heme content present in the nucleus. Furthermore, comparison of the different ratios of EGFP/mKATE2 fluorescence intensities between the 2 heme sensors in all 4 compartments points to rather similar, but somewhat higher, free heme pools in chloroplasts and mitochondria relative to those in the cytoplasm and the nucleus.

### Heme accumulation in subcellular compartments is enhanced by feeding with ALA

In order to demonstrate the suitability of the heme sensor for the evaluation of varying relative levels of free heme, the cellular heme content was systematically altered using several approaches. First, we added 5-aminolevulinic acid (ALA) to leaf discs obtained from tobacco plants (*Nicotiana benthamiana*) that transiently expressed one or other of the heme sensors. We applied ALA in a dose-dependent manner in order to verify that the EGFP fluorescence was indeed correlated with the amounts of synthesized and subcellularly available heme. After transient transformation with gene constructs encoding the heme sensor variants by infiltration of leaves, the leaves were incubated in the presence of different concentrations of ALA (0 mm = mock solution, 0.5, 1, and 2 mm ALA).

**Figure 2.**
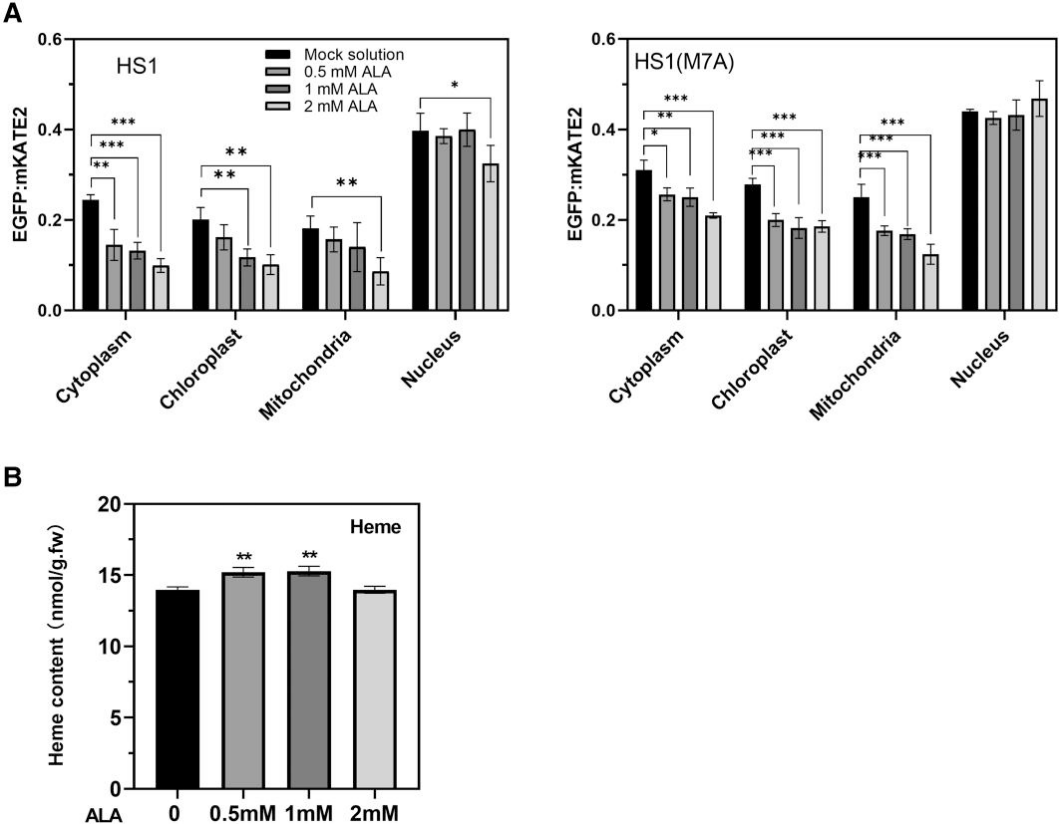
EGFP/mKATE2 fluorescence ratios recorded by the 2 heme sensors HS1 and HS1(M7A) in different cellular compartments of transiently transformed *N. benthamiana* leaves following supplementation with exogenous ALA. *N. benthamiana* leaves were transiently transfected with genes encoding HS1/HS1(M7A) variants targeted to different subcellular compartments (chloroplasts, mitochondria, nuclei, and cytoplasm) and infiltrated with different concentrations of ALA solution (0, 0.5, 1, and 2 mm). **A)** The ratio of EGFP to mKATE2 fluorescence emitted by the 2 heme sensor variants was determined after treatment with different concentrations of ALA. The ratio of the EGFP to mKATE2 fluorescence is calculated based on the sum of pixels in the EGFP and mKATE2 channels. Images show the fluorescent signal of chloroplasts, the fluorescent signals from the HS1 variants (EGFP domain and mKATE2 domain) in different cellular compartments (cytoplasm, chloroplast, mitochondria, nucleus), and the merged fluorescence signals of EGFP and mKATE2. For transient transformation, 6-wk-old leaves are used. Leaves of the same age were transfected and processed after 48 h of ALA treatment. The EGFP/mKATE2 ratios obtained from the EGFP and mKATE2 channels were calculated from 5 to 8 transformed representative cells of different transformants. Statistical significance of different cellular compartments compared with the fluorescence ratio in the control group (mock solution) is indicated by Tukey's HSD method (*P < 0.05; **P < 0.01), and error bars represent the sd of 3 biological replicates. **B)** Total heme content of leaf material. For HPLC analyses, leaves from 6-wk-old plants were used. Statistical significance compared with the control (mock solution) is indicated by Student's *t*-test (*P* < 0.05), and error bars represent the sd of 3 biological replicates.

Compared with the control, stepwise addition of increased levels of ALA to leaf discs correlated in all subcellular compartments with a reduction in the EGFP/mKATE2 ratio of the fluorescent signal, which is indicative of elevated levels of subcellular free heme (Fig. 2). However, the heme pool in the nuclei is markedly less responsive to supplementation with ALA up to 1 mm, as the EGFP/mKATE2 signal ratios did not change significantly with increasing amounts of ALA. However, in all other cellular compartments, the gradual increase in added ALA led to a stepwise decrease in the EGFP/mKATE2 fluorescence signal ratio. In comparison with the data for HS1(M7A), the effect of gradual decreased EGFP/mKATE2 ratios was more pronounced in plastids and mitochondria when the HS1 sensor was used following ALA feeding. This observation points to the improved suitability of HS1 relative to that of HS1(M7A) and a high gradual decrease of the EGFP/mKATE2 fluorescence signal ratio in plastids and mitochondria. Gradually declining ratios of the EGFP/mKATE2 fluorescence signal were also observed in the cytoplasm when HS1 and HS1(M7A) were used to sense the free heme pool in the cytoplasm. The drastic drop in the fluorescence signal ratio reported by HS1 for the cytoplasm in ALA-supplemented leaves, relative to the ratio after treatment with mock solution, highlights the marked impact on the free heme pool, when excess ALA is available and is most probably efficiently converted into a surplus of heme, which is then released from plastids into the cytoplasm. Thus, it is assumed that a rise in the heme pool in the cytoplasm as a result of altered heme metabolism in plastids (e.g. owing to enhanced heme synthesis as result of ALA feeding) is effectively detectable with the HS1 variant in comparison with the application of the transiently expressed HS1(M7A) mutant, because the EGFP/mKATE2 signaling ratio changed more clearly due to the relatively high heme content in the cytoplasm.

Moreover, as expected, these results suggest that the cytoplasmic pool of free heme increases in response to increasing levels of added ALA to a similar extent in plastids and mitochondria. As this alteration in the fluorescence ratio of EGFP/mKATE2 correlates with enhanced ALA supply, the heme sensors indeed provide a rather accurate picture of the changes in the availability of free heme during modulated heme metabolism and most likely also modified intracellular heme sensing. Measurements of the total amount of noncovalently bound heme in leaves of *N. benthamiana* in which the HS1 variants were transiently expressed also revealed a slight increase in the heme content after 2 d of feeding with ALA (up to 1 mm). The increased supply of ALA (2 mm) caused a slight drop in noncovalently bound heme, which might be explained by an inhibitory feedback effect on heme formation. However, we are aware that our heme analysis of the leaf extracts must also take into account the amount of heme contributed by the agrobacteria used for transient transformation.

### Inhibition of ALA synthesis reduces levels of subcellular free heme

**Figure 3.**
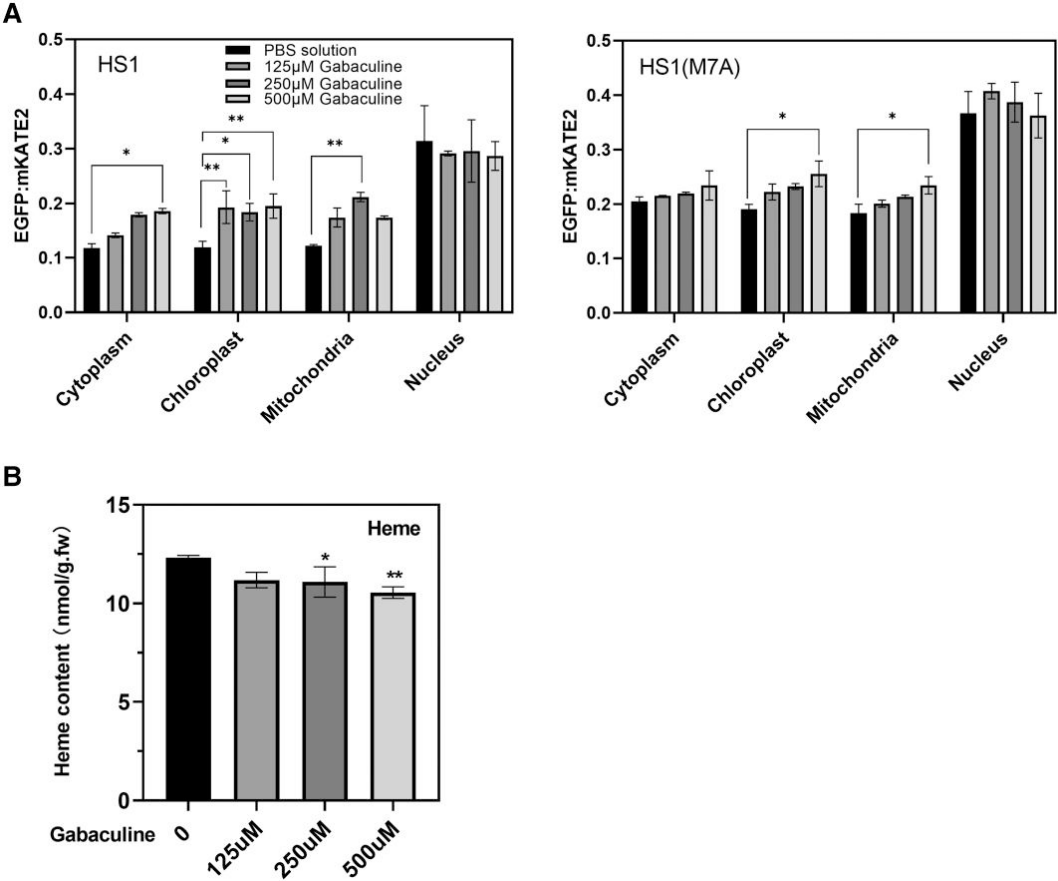
Fluorescent signals and their EGFP/mKATE2 ratios recorded by the 2 heme sensor variants HS1 and HS1(M7A) in different cellular compartments of transiently transformed *N. benthamiana* after incubation of leaves with gabaculine. Transiently transformed tobacco leaves were exposed to different concentrations (0, 125, 250, and 500 *µ*m) of gabaculine in a phosphate-buffered saline (PBS) buffer for 24 h. **A)** EGFP and mKATE2 fluorescence ratios recorded by HS1(M7A) and HS1 in the different cellular compartments of *N. benthamiana* leaves that had been treated with different concentrations of gabaculine. The fluorescence ratio of EGFP/mKATE2 is calculated from the sums of pixels in the EGFP and mKATE2 channels. Statistical significance of different cellular compartments compared with the fluorescence ratio in the control group (PBS solution) is indicated by Tukey's HSD method (*P* < 0.05), and error bars represent the sd of 3 biological replicates. **B)** Statistical significance compared with the control (PBS solution) is indicated by Student's *t*-test (*P* < 0.05), and error bars represent the sd of 3 biological replicates.

Alternatively, free heme content can be reduced by inhibition of the TBS pathway. This was specifically achieved by applying gabaculine (Hoober et al. 1988), which inhibits glutamate-1-semialdehyde aminotransferase, the second enzyme in the ALA synthesis pathway (Grimm 1990). We added different levels of gabaculine to wild-type *N. benthamiana* cells that expressed either the high-affinity heme sensor HS1 or the medium-affinity sensor HS1(M7A) and measured levels of both total and free heme. As demonstrated in Fig. 3, inhibition of ALA synthesis upon incubation of leaf discs with gabaculine results in an increase in the EGFP/mKATE2 fluorescent signal ratio, from 1.90 (±0.09) to 2.82 (±0.04). These data are consistent with a decrease in heme binding to the sensor and diminished free heme content. Thus, it is predicted that the relative amount of free heme in each compartment can be gradually modified by the dose-dependent supply of gabaculine. However, once again, increasing gabaculine inhibition had little or no effect on the content of free heme in the nucleus. With the exception of plastids, the gradual decrease in the levels of free heme in mitochondria and the cytoplasm can be more accurately determined with the aid of HS1, but the expression of HS1(M7A) also reliably detects changes in the ratio of the EGFP/mKATE2 fluorescence signal in the analyzed cellular compartments (Fig. 3). Analysis of the total amount of heme in transfected leaf cells indicates a slow decrease, which implies that the free heme pool in all cellular compartments determined is more strongly affected by inhibition or inactivation of plastid-localized ALA synthesis. The free heme level decreased, as indicated by a higher fluorescence ratio of EGFP to mKATE2.

In summary, the EGFP/mKATE2 fluorescence ratios increase when either TBS or heme accumulation is inhibited. This corresponds to a decrease in the rate of accumulation of free heme (Fig. 3). In addition to the decrease in free heme content, inhibition of ALA synthesis by gabaculine leads to a slight reduction in the overall content of noncovalently bound heme (Fig. 3B).

### Analysis of free heme during development

Subcellular levels of free heme are also significantly modified in young and old leaves that were transiently transformed with the heme sensor gene constructs. Young tissue clearly shows a lower subcellular EGFP fluorescence signal relative to that of mKATE2 in chloroplasts, and to a lesser extent in also the cytoplasm, while the mitochondria and nuclei showed no change in this parameter between young and old tissues (Supplementary Fig. S1, A and B). Lower EGFP fluorescence in young plastids is indicative of elevated activity of TBS and higher availability of free heme than in older tissue. It is well known that emerging or greening seedlings and developing leaves have enhanced ALA and TBS compared with older plant tissues, in which TBS is mainly required for turnover and recycling of heme and chlorophyll-dependent proteins. The lower steady-state level of heme is also confirmed by measurements of the total noncovalently bound heme content in cells (Supplementary Fig. S1C).

We also applied different concentrations of hemin (500 *µ*m and 1 and 2 mm) to leaf discs obtained from transiently transformed *N. benthamiana* plants, but we could not detect any significant change in the free heme content of any of the subcellular compartments—not even in the cytoplasm, the cellular compartment immediately adjacent to the extracellular space, which is separated from it only by the plasma membrane (Supplementary Fig. S2). Apparently, hemin uptake into leaf cells is inefficient in the absence of additional surfactants.

### Sensing of free heme in *Arabidopsis* and tobacco after stable transformation with heme sensor genes

**Figure 4.**
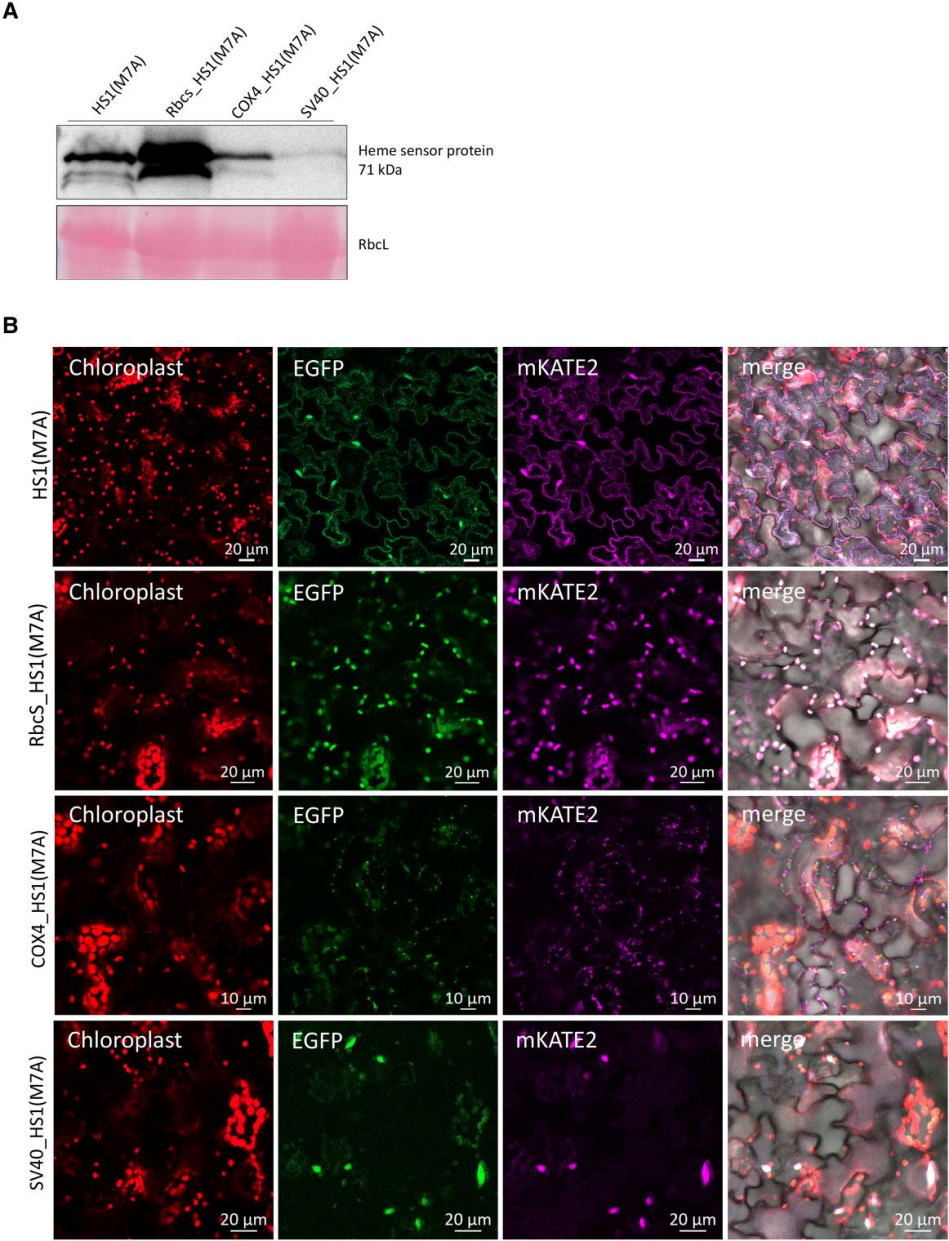
Quantification of free heme by the heme sensor HS1(M7A) in subcellular compartments of stably transformed *A. thaliana.* Transformants of *Arabidopsis* plants stably expressing HS1(M7A) in subcellular compartments are used here as an example. The heme sensor sequences were ligated with 2 transit peptides, RbcS and COX4, for transport from the cytoplasm to the chloroplasts and mitochondria and with a nuclear localization signal SV40, which mediates the transport of proteins from the cytoplasm to the nucleus. **A)** Immunoblotting of the heme sensor protein in different cellular compartments of stably transformed *A. thaliana* lines. The EGFP antibody was used. **B)** The fluorescence signal of HS1(M7A) in different cellular compartments. The images show the fluorescence signal of the chlorophyll in the chloroplasts; the heme sensor expressed in the cytoplasm, chloroplasts, mitochondria, and nucleus (fluorescence of the EGFP domain and mKATE2 domain, respectively); and the merging of the abovementioned fluorescences.

**Figure 5.**
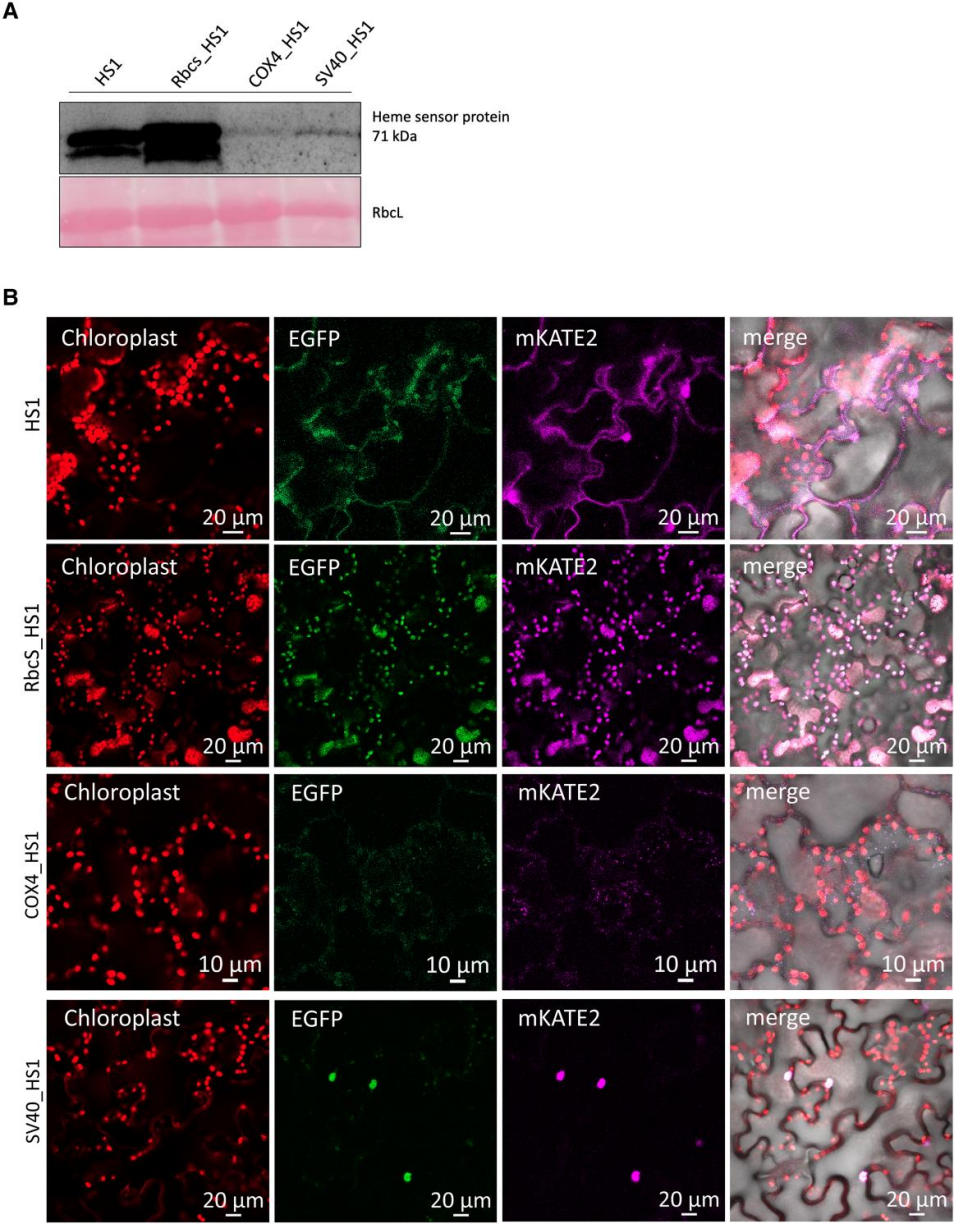
Quantification of free heme by the heme sensor HS1 in subcellular compartments of stably transformed *N. tabacum.* The *heme sensor* coding sequences were ligated with the coding sequences of the transit peptides of RbcS and COX4 and the nuclear localization signal SV40. These transgenes were introduced into the tobacco genome. The fluorescence ratios of EGFP and mKATE2 of HS1 are shown as examples for selected transgenic lines. **A)** Immunoblotting of the heme sensor protein in different cellular compartments of stably transformed *N. tabacum* lines. **B)** The fluorescence signal of HS1 in different cellular compartments. The images show the fluorescence signal of the chlorophyll in the chloroplasts; the heme sensor expressed in the cytoplasm, chloroplasts, mitochondria, and nucleus (fluorescence of the EGFP domain and mKATE2 domain, respectively); and the merging of the abovementioned fluorescences.

Finally, we generated stable transformants of *Arabidopsis* (*Arabidopsis thaliana*) and tobacco (*Nicotiana tabacum*) expressing transgenes encoding one or other of the 2 heme sensor variants for translocation in the 4 cellular subcompartments, in order to monitor changes in heme availability. The accumulation of transgenic heme sensor proteins was confirmed by immunoanalysis. Normalized to the fresh weight of the leaf, the 71-kDa heme sensors accumulated to different extents in the different cellular subcompartments. However, each transgenic protein product accumulates in fairly similar amounts relative to the volume of each of the 4 cellular compartments (Figs. 4A and 5A). The stably transformed *Arabidopsis* and tobacco lines did not show any aberrations in development, growth rate, green pigmentation, or leaf size (Supplementary Figs. S3 and S4). Always 2 lines were selected for immune analysis of the 2 heme variants in the 4 different cellular compartments, cytoplasm, plastids, mitochondria, and nuclei. The immunoblot revealed similar amounts of the HS1 variants in each line, which contains the same transgene, but the accumulation of the heme sensor differed in each cellular compartment (Supplementary Fig. S5). It can therefore be assumed that heme binding to the transgenic heme sensor does not impair heme metabolism or general viability. This is consistent with the earlier observations, which showed that heme binding to HS1 remains reversible, and expression of the heme sensor in yeast cells does not itself perturb heme homeostasis or otherwise affect viability (Hanna et al. 2016). The fluorescence signal reported by the HS1 variants is always exclusively detectable in the correct subcellular compartment (Figs. 4B and 5B). We evaluated whether different expression levels of the transgenic HS1 genes, leading to different amounts of accumulating HS1 proteins, have any influence on the ratio of EGFP to mKATE2 fluorescence under conditions in which similar amounts of free heme are expected in any given cellular compartment in different transgenic lines. As an example, 4 different lines were selected, which express RbcS_HS1(M7A) in different amounts. On average, the EGFP to mKATE2 ratio appeared to be similar in each of the 4 independent transgenic lines (for each line, at least 3 different progenies were analyzed; see Supplementary Fig. S6). Thus, we conclude that irrespective of the level of the heme sensor expressed, the fluorescence of EGFP relative to that of mKATE2 always (inversely) reflects the relative amount of free heme present in the stable transformants expressing either of the HS1 variants. Although different lines express each of the different *HS1* gene constructs to different extents, the amount of bound heme corresponds to the relative level of free heme.

ALA feeding was also conducted on leaves from transgenic *Arabidopsis* lines that stably express and translocate one of the 2 HS1 variants into one or other of the 4 subcellular compartments. Leaves of the same transgenic line were incubated with either 0, 0.5, 1, or 2 mm ALA solution for 24 h, and 3 independent lines for each gene construct were selected as representative of other experiments with other transgenic lines. The EGFP and mKATE2 signals reported by HS1(M7A)-expressing *Arabidopsis* lines after incubation of their leaves are listed in Supplementary Fig. S7. The EGFP/mKATE2 ratio of their fluorescence signals in the 3 different compartments—chloroplasts, mitochondria, and nucleus—of the respective transgenic lines was determined and revealed an increase in the content of free heme in chloroplasts and mitochondria under the influence of excess ALA supply, while the heme levels in the nucleus were relatively stable (Supplementary Fig. S7). In addition to the increase in free heme, the total heme content, which is based on the measurement of noncovalently bound heme, remained constant in the HS1(M7A)-expressing lines. Similarly, leaves of the stable transformants were also treated with gabaculine, and the ratios of EGFP/mKATE2 fluorescence signals were determined after 24 h of incubation. The images of the fluorescence microscope show for the transgenic lines a slight increase in the EGFP to mKATE2 fluorescence ratio in the 2 organelles chloroplast and mitochondrion, which corresponds to a slight loss of free heme with increasing concentration of the inhibitor. The content of free heme in the cell nuclei again appears to be generally lower than in the plastids and mitochondria and was not altered by the inhibition of ALA synthesis by gabaculine (Supplementary Fig. S8).

### The fluorescence signals reported by HS1 and HS1(M7A) correlate with heme content in both wild-type and AtFC1-overexpressing *N. tabacum* plants

To test the applicability of HS1 for the determination of free heme levels in the various cell compartments of mutant and transgenic lines, we transiently expressed the heme sensor in *N. tabacum* (cv. ‘SNN’) wild-type and transgenic plants that overexpressed recombinant *Arabidopsis* ferrochelatase1 (AtFC1) (Supplementary Fig. S9) and compared the EGFP/mKATE2 ratios of both tobacco lines. The results revealed that the pool of free heme in the cytoplasm of AtFC1-expressing plants was larger than that of the wild type, while the opposite was observed in the chloroplasts of both lines. These observations can be explained by the differences in function and localization between FC1 and the FC2 isoform (Fan et al. 2019). FC1 accumulates in the stroma of plastids, is associated with the envelope membrane, and serves the need for extraplastidal heme, while the thylakoid-localized FC2 is responsible for the provision of heme of heme-dependent proteins in plastids (Fan et al. 2019, 2023; Hedtke et al. 2023). Excess AtFC1 may compete for substrate with the endogenous FC2, which may result in a significant accumulation of heme in the cytoplasm.

## Discussion

### The dynamics of heme metabolism determine the availability of heme for cellular processes

Efforts have been made to predict the numbers of heme-dependent proteins in the proteomes of prokaryotes and eukaryotes by using bioinformatic tools or mass spectrometric approaches (Shimizu et al. 2020; Wilkinson et al. 2023; Kim et al. 2023). However, even though they represent one of the most crucial groups of proteins for vitality and survival under adverse environmental growth conditions, very few quantitative analyses have been undertaken, in particular of the relative amounts of these proteins found in diverse subcellular compartments. This applies above all to the heme-dependent proteins in plants (Richter et al. 2023). Furthermore, while efforts to quantify noncovalently bound heme in plant tissues have been reported (Thomas and Weinstein 1990; Masuda and Takahashi 2006; Espinas et al. 2012, Espinas et al. 2012), owing to technical limitations and methodological shortcomings, no reliable analyses of subcellular heme contents in the subcellular compartments of plants have been reported to date, especially of the free or labile (i.e. available) heme.

The constant demand for adequate amounts of heme in all subcellular compartments of plant tissues suggests highly flexible synthesis of heme, tight control of its metabolism, and dynamically variable provision of free heme. In each plant organ (root, shoot/leaf, and flower), plastidic heme synthesis must be flexibly adjusted to the requirements of the cellular subcompartments, also in view of the dependence of plants on appropriate developmental and environmental conditions. Plants possess 2 genes for the heme-synthesizing enzyme ferrochelatase, and it has been proposed that FC2, whose hydrophobic CAB domain projects into the thylakoids, is responsible for the synthesis of heme for plastidic heme-dependent proteins, whereas FC1, as a water-soluble isoform that is associated with the envelope membrane, supplies heme to heme-dependent proteins outside the plastids (Espinas et al. 2016; Fan et al. 2019, 2023). While heme synthesis and its subcellular allocation ensure its bioavailability for heme-dependent processes, its overaccumulation must be avoided, owing to its cytotoxicity. Thus, amounts of freely available heme are expected to fluctuate in accordance with the temporally and spatially varying demands for this prosthetic group.

### Transiently expressed heme sensors can be used to estimate the relative amount of free heme

The availability of heme must be controlled at the level of free heme, which is indispensable for rapid subcellular molecular responses. Thus, organisms are thought to have a reliable pool of labile heme, which allows the compound to be mobilized for use in all subcellular compartments (Hanna et al. 2018; Donegan et al. 2019). This explains the need for a lowly invasive method for the quantification of the subcellular levels of free heme, and HS1, a heme-sensing fusion protein consisting of 2 fluorescent peptide domains and a cytochrome, has been presented for this purpose (Hanna et al. 2016). Numerous heme sensor proteins have been published that make use of fluorescently labeled fusion proteins with heme-binding capacity to determine levels of free heme (Takeda et al. 2003; Briand et al. 2012; Song et al. 2015; Taira et al. 2015; Abshire et al. 2017; Wißbrock and Imhof 2017; Bairwa et al. 2020; Leung et al. 2021). We chose HS1 and its lower-affinity variant because of the ease of expression of HS1 in different plant cell compartments and the apparent reliability of the ratiometric principle for determining the relative amount of heme using the 2 fluorescent proteins in the HS1 fusion construct.

We wanted to evaluate the suitability of the sensor HS1 and its less sensitive derivative HS1(M7A) for the quantification of the relative amounts of free heme in the subcellular compartments of plants. The stable accumulation of HS1 and its isoform HS1(M7A) was confirmed after transient and stable transformation of gene constructs encoding the transgenic HS1 variants with transit and signal sequences for translocation into plastids, mitochondria, nucleus, and cytoplasm (Fig. 1A), and the detectability of both EFGP and mKATE2 fluorescence was verified (Fig. 1). We then confirmed that variations in heme metabolism could also be verified based on the changing ratio between the EGFP and mKATE2 fluorescence signals, which is inversely proportional to the levels of free heme pools in the cytoplasm and organelles. Since the EGFP/mKATE2 ratio of the fluorescence signal in the plastids was always lower than in the other compartments analyzed, it is assumed that plastids generally accumulate more free heme than other subcellular structures (Fig. 1).

Using transient expression of the HS1 protein in *N. benthamiana*, we monitored the changes in subcellular heme contents including the free heme pool in response to either supplementation with the heme precursor ALA (Fig. 2) or inhibition of ALA synthesis by incubation of leaves with gabaculine (Fig. 3). ALA synthesis is considered to be the rate-limiting step and is tightly controlled by various posttranslational mechanisms, including heme feedback control (Wang et al. 2022). With increasing ALA supply, the ratio of EGFP to mKATE2 fluorescence decreased in all compartments except the nucleus, although the differences between these ratios were more pronounced when the HS1(M7A) variant was expressed (Fig. 2). We also attempted to infiltrate leaf discs from transformed *N. benthamiana* plants with hemin, but no changes in the ratio of EGFP to mKATE2 fluorescence signal were observed following the exogenous supply of increasing amounts of hemin. Measurements of the total heme contents of leaf discs incubated with hemin-containing solutions only led to ambiguous and nonreproducible results. We often observed a more reliable difference in the EGFP/mKATE2 fluorescence ratios when HS1 is applied in the subcellular compartments after the incubation with exogenous ALA, but we also confirmed the efficacy of the HS1(M7A) variant with its lower heme-binding affinity in chloroplasts. Then, the EGFP/mKATE2 fluorescence signal ratio of HS1(M7A) rather than that of HS1 shows a higher heme content in chloroplasts relative to the other subcellular compartments, as mentioned in the previous paragraph.

Variations in ALA synthesis rate can be observed in many mutants and transgenic plants with mutation of genes involved in the highly regulated steps of TBS, i.e. ALA synthesis itself, magnesium (Mg) chelation, and the reduction of protochlorophyllide (for review, see Wang et al. 2022). Changes in the steady-state contents of heme in plastids and in the cytoplasm can also affect the synthesis of chlorophyll and heme by feedback control. Thus, it has been proposed that heme-mediated signaling affects the transcriptional control of photosynthesis-associated nuclear genes (PhANGs; Woodson et al. 2011), which includes light-induced genes that are involved in TBS, such as *HEMA* (encoding glutamyl-tRNA reductase [GluTR]), *CHLH* (encoding the H-subunit of Mg chelatase), or *CAO* (encoding the chlorophyll a oxygenase). Plastidic heme has a negative feedback effect on ALA synthesis. When heme binds to GluTR-binding protein (GBP), it destabilizes the interaction between GBP and GluTR (Richter et al. 2019). The HS1 protein provides a useful tool to monitor the impact of free heme inside and outside the plastids. These heme pools may act either upon the transcriptional control of nuclear genes or posttranslationally on ALA synthesis in chloroplasts. Heme-dependent control is thought to be promoted and controlled by changes in the levels of free heme, which are expected to be modified in response to deregulated ALA synthesis in mutants and transgenic plants.

Initially, we were not sure whether it could be ruled out that overexpression of HS1 combined with a high binding capacity of free heme could have a regulatory effect on heme metabolism, e.g. if HS1 binding of heme were to perturb TBS and lead either to a lack of free heme or stimulate heme synthesis in response to a deficiency of free heme. In such a case, the EGFP/mKATE2 fluorescence ratio might not necessarily correspond to the wild-type content of free heme, but a consequence of deregulated TBS, most likely at the level of ALA synthesis. However, all transgenic lines analyzed so far, in which HS1 derivatives are expressed in one of the subcellular compartments, show wild-type-like phenotypes and no deviation in chlorophyll and heme content (Supplementary Figs. S3 and S4). Based on our observations, we propose that the binding of free heme by HS1 does not affect the cellular heme metabolism.

### Stable transformation of the different variants of the *HS1/HS1(M7A)* genes enables research on heme homeostasis

After stable or transient transformation with the *HS1* or *HS1(M7A)* gene, and the isolation of individual transformants, the heme sensor variant probably accumulates to varying extents in the different subcellular compartments of each of the different lines. Nevertheless, steps must be taken to ensure that the determined ratio of EGFP to mKATE2 fluorescence in each line of a transformation series expressing the same heme sensor construct but at different level reflects the relative free heme content. Using different transgenic *Arabidopsis* lines with different expression intensities of the *RbcS_HS1(M7A)* gene, similar EGFP/mKATE2 ratios were determined for plastid-located fluorescence of the heme sensor (Supplementary Fig. S6). For a given affinity of the heme sensor for heme, it can be deduced that the relative amount of accumulating heme sensor variants always correlates with the relative amount of free heme to which the sensor protein is bound. In other words, a highly expressed HS1 binds larger amounts of heme, and a smaller amount of the heme sensor binds less free heme from the same heme pool. It will always result in a fluorescence ratio that reflects a certain relative amount of free heme. The HS1-bound amount of heme therefore always corresponds to the relative size of the free heme pool, at least for a given range of *HS1* transgene expression.

**Figure 6.**
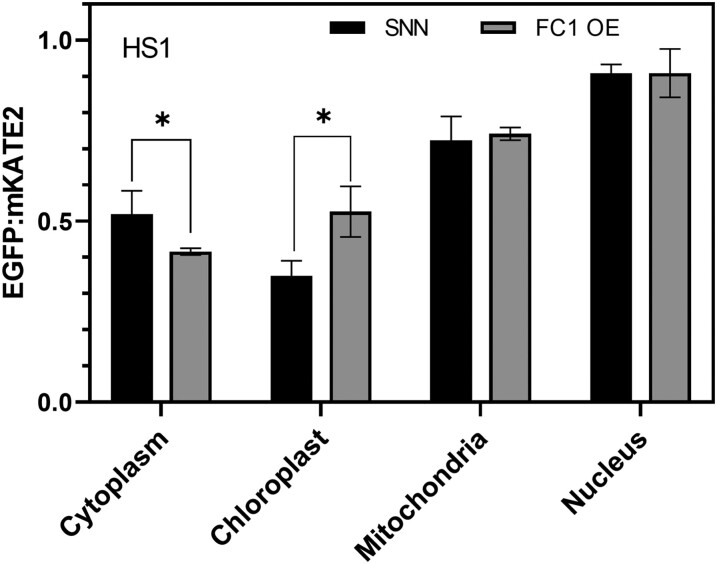
GFP/mKATE2 fluorescence ratio recorded after transient expression of the heme sensor HS1 in wild-type *N. tabacum* and AtFC1-overexpressing plants. The EGFP/mKATE2 fluorescence ratios reported by HS1 for the various subcellular compartments of wild-type *N. tabacum* (SNN) leaves and the *AtFC1*-overexpressing (FC1 OE) line are shown in the graph. The ratio is derived from the sum of the numbers of pixels of EGFP and mKATE2 fluorescence. Note that the ratio is inversely proportional to the level of free heme present. Statistical significance of different cellular compartments compared with the fluorescence ratio in the WT plants (SNN) is indicated by Tukey's HSD method (*P* < 0.05), and error bars represent the sd of 3 biological replicates.

In stably *HS1*-expressing lines of *Arabidopsis*, the relative amounts of free heme determined in 1 cell compartment of the plants can now be used for comparative studies of samples from plants that have either been pharmacologically treated, e.g. by addition of precursor molecules of TBS (e.g. ALA feeding; Supplementary Fig. S7), originate from different tissues, represent other developmental stages (Supplementary Fig. S1), or are derived from mutants or transgenic lines in the same developmental state as the reference sample (Fig. 6). The levels of free heme always represent only a small proportion of the total heme content, which consists of heme that is either covalently or noncovalently bound to heme-binding proteins, but free heme is expected to vary dynamically, depending on the immediate metabolic demand and the need for protection from stress and for signaling purposes (Vavilin and Vermaas 2002; Espinas et al. 2012). Thus, since the free heme pool reacts more sensitively to intracellular perturbations and exogenous stimuli, the HS1 sensor is a useful tool for the accurate analysis of the subtle regulatory impact of free heme on metabolism and cell function. Alterations of heme levels are to be expected in mutants of TBS (Fig. 6), chloroplast biogenesis, photosynthesis, responses to abiotic and biotic stresses, nitrogen fixation, and formation of nodules. Experiments that aim to clarify the regulation and provision of transporters and factors for the necessary supply of heme to the extraplastidal spaces of the plant cell are also likely to be important in this context. How heme is transported from the plastidal site of heme synthesis into the cytoplasm and other organelles and subcellular compartments remains one of the great unknowns in plant cell biology. The range of applications for heme sensors in the plant area is therefore likely to increase rapidly in the near future.

Future work may also elucidate the extent to which the 2 HS1 variants are differentially loaded with heme in the different subcellular compartments of *Arabidopsis* Col-0 ecotype. To what percentage the accumulating HS1 proteins can be loaded with heme remains an open question. It will also be interesting to verify the heme-loading capacities of the 2 HS1 variants in different *Arabidopsis* ecotypes or in other plant species, and this would make it possible to find out to what extent the quantities of free heme in the cells of different plant species differ. It could also be examined whether the affinity of ferric and ferrous heme differs in the subcellular compartments of plant cells.

## Conclusion

In this initial attempt to estimate relative amounts of free heme in plant organelles, we have successfully used 2 previously reported heme sensors, HS1 and HS1(M7A), and obtained data for 4 different subcellular compartments. We confirmed the ability of these sensors, when genetically introduced to plant cells, to assess free heme levels and verify that they differ in response to pharmacological disruption and genetic alterations of heme metabolism. The method opens up many other possibilities to monitor the dynamics of free heme and its mobilization. These include tracking the relationship between free heme and total heme, as well as heme metabolism, redistribution, and signaling in plants. Using HS1 transiently or stably expressed in plant tissue, it should be possible to determine the actual amounts of bioavailable heme after normalization of levels.

## Materials and methods

### Preparation of the heme sensor gene constructs

The *HS1* (*yHS1*) and *HS1(M7A)* (*yHS1-M7A*) gene constructs (Hanna et al. 2016), which were controlled by the 35S-CaMV promoter, were used for subcloning transgenes for targeting of the heme sensor variants into the plant subcellular compartments cytoplasm, nucleus (using the SV40 nucleation coding sequence), mitochondria (using the *COX4* transit peptide coding sequence), and plastids (using *RbcS* transit peptide coding sequence) (Fig. 1A; specific primer pairs in Supplementary Table S1). The amplified transgene constructs were inserted into the binary pGL1 vector that was opened with XbaI and SacI. All PCR amplifications were performed using the S7 Fusion Polymerase (Biozym Scientific GmbH, Germany). The final vectors were transformed into the *Agrobacterium tumefaciens* strain GV 2260 for transient and stable transformation.

### Plant growth condition and transformation

Five-week-old *N. benthamiana* plants grown under long-day conditions (16-h light/8-h dark cycles, 100 *μ*mol photons m^−2^s^−1^ light intensity, 23 °C, 50% relative humidity) were transiently transformed with one or other of the 8 different gene constructs encoding the different heme sensor variants (Fig. 1A) and incubated in darkness for 3 d. Wild-type *Arabidopsis* (*A. thaliana*) plants (ecotype Col-0) were grown under short-day conditions (8-h light/16-h dark cycles) and a light intensity of 100 *μ*mol photons m^−2^s^−1^ at 23 °C and 60% relative humidity and stably transformed by dropping aliquots of the *A. tumefaciens* cell suspension onto emerging flowers, which were then incubated in the dark for 2 d, before being transferred into the light and grown until seed ripening. Germinating transgenic seeds were selected on plates containing the herbicide BASTA, and homozygous T3 progenies were selected for further studies. Leaf discs of sterile young *N. tabacum* leaves were transformed with the transgenic *Agrobacterium* strains, and transgenic lines were first selected on BASTA and then on cefotaxime-containing 2MS medium, prior to the detection of their fluorescent proteins by fluorescence microscopy.

### DNA extraction and transgene analysis

Leaf samples of transgenic lines of *A. thaliana* and *N. tabacum* were harvested and homogenized in 100 *μ*L of DNA extraction buffer (200 mm Tris pH 8.0, 25 mm EDTA, 150 mm NaCl, and 0.5% SDS [*w/v*]), and 80-*μ*L aliquots were centrifuged for 5 min (15,000 × *g*, 4 °C). DNA was precipitated from the supernatant and subjected to genotyping (see primers in Supplementary Table S1) using Taq DNA polymerase (Thermo Fisher Scientific, Waltham, MA, USA) for PCR.

### Protein extraction and immunoblot analysis

Leaf samples of single *Arabidopsis* and *N. tabacum* seedlings were homogenized in liquid nitrogen and incubated in 200 *μ*L of PEB buffer (2% SDS, 12% sucrose, 56 mm NaCO_3_, and 2 mm EDTA, pH 8.0) at 70 °C for 20 min and centrifuged (15,000 × *g*, room temperature (RT) 10 min). Equal amounts of the supernatants from each sample were applied to a 12% SDS–PAGE gel as described before and analyzed by immunoblotting using the anti-GFP antibody (dilution 1:2,000, detection by SuperSignal West Pico Chemiluminescent substrate [Thermo Fisher Scientific]).

### HPLC analysis of heme

Homogenized samples of *Arabidopsis* and *N. tabacum* leaves were extracted in acetone/0.2 m NH_4_OH and centrifuged (15 min), and the pellets were dissolved in 200-*μ*L acetone/hydrochloric acid/DMSO (AHD, 10:0.5:2, *v/v/v*). After centrifugation (twice for 15 min each) at 15,000 × *g* and RT, the supernatant was subjected to HPLC analysis on an Agilent 1100 or 1290 HPLC system equipped with a diode array and fluorescence detectors (Agilent Technologies). The Agilent ChemStation (product no. G2170BA) was used for HPLC analyses. Quantification was conducted with hemin standards.

### ALA, gabaculine, and hemin treatments

Mock solution (20 mm Tris, pH 7.4) was supplemented with different concentrations of ALA (0.5, 1, and 2 mm for ALA feeding), gabaculine (0.5, 1, and 2 mm, for inhibition of ALA synthesis), or hemin (125, 250, and 500 *μ*m). Leaf samples were incubated for up to 24 h in darkness, dried and frozen in liquid nitrogen, or directly examined by confocal microscopy.

### Fluorescence imaging and fluorescent ratio calculation

Fluorescence detection and image acquisition were performed using the confocal microscope LSM 800 (Carl ZEISS Microscopy GmbH, Germany). Leaf tissue was placed on a glass slide dripped with buffer, covered with the slide and ensuring no air bubbles upon the leaf slice, observed under the confocal laser scanning microscope (EGFP: excitation wavelength 490 nm, emission wavelength 510 nm; mKATE2: excitation wavelength 590 nm, emission wavelength 630 nm). The pixel sum of mentioned channels in the selected area of each individual fluorescent acquisition was used for the calculation (relative free heme content can be shown by pixel sum of EGFP/pixel sum of mKATE2 100%). Fluorescence ratio of EGFP/mKATE2 of HS1(M7A) and HS1 in the different cellular compartments of the analyzed plant species was obtained from GFP and KATE2 channels and calculated from more than 5 to 8 transformed representative cells of each transformant.

### Statistical analyses

Statistical significance compared either with the respective control or with the fluorescence ratio of the respective control is indicated by Tukey's HSD method (*P* < 0.05). Error bars represent the sd of the 3 biological replicates.

## Supplementary Material

kiae291_Supplementary_Data

## Data Availability

All relevant biological materials are available from the corresponding author upon request. Supporting data are available in the Supplementary data published online.

## Supplementary data

The following materials are available in the online version of this article.

**Supplementary Figure S1.** Fluorescent signals and EGFP/mKATE2 ratios reported by the heme sensors HS1 and HS1(M7A) following the transient expression of each in old and young leaves of *N. benthamiana*.

**Supplementary Figure S2.** Fluorescent signals and GFP/mKATE2 ratios of HS1(M7A) in different cellular compartments of transiently transformed *N. benthamiana* after a treatment with hemin.

**Supplementary Figure S3.** Image of *A. thaliana* wild-type and transgenic lines expressing the 2 heme sensor variants HS1 and HS1(M7A).

**Supplementary Figure S4.** Image of 20-d-old *N. tabacum* wild-type and transgenic lines expressing the heme sensor HS1 in different subcellular compartments.

**Supplementary Figure S5.** Immune analysis of the heme sensor variants HS1 and HS1(M7A) in stably transformed *A. thaliana* and *N. tabacum* lines.

**Supplementary Figure S6.** Determination of the EGFP/mKATE2 ratio reported by HS1(M7A) in transgenic tobacco lines.

**Supplementary Figure S7.** Fluorescence signals of the heme sensor protein and ratio of the EGFP/mKATE2 fluorescence signal of 3 different HS1(M7A) stably transformed 4-wk-old *Arabidopsis* lines after treatment with ALA for 24 h.

**Supplementary Figure S8.** EGFP/mKATE2 ratios reported for 3 different stably transformed 4-wk-old *Arabidopsis* lines that had been treated with the TBP inhibitor gabaculine for 24 h.

**Supplementary Figure S9.** Image of seedlings of a representative AtFC1 overexpression line and wild type of *N. tabacum*.

**Supplementary Table S1.** Primers used for the subcloning of the gene constructs encoding either the HS1 or the variant HS1(M7A).

## Dive Curated Terms

The following phenotypic, genotypic, and functional terms are of significance to the work described in this paper:

CAO Gramene: AT2G47450
CAO Araport: AT2G47450

heme CHEBI: CHEBI:30413

dna Gramene: EO:0007532
dna Araport: EO:0007532

EDTA Gramene: Ethylenediamine tetraacetic acid
EDTA Araport: Ethylenediamine tetraacetic acid

GBP Gramene: AT3G21200
GBP Araport: AT3G21200

CHLH Gramene: AT5G13630
CHLH Araport: AT5G13630

## References

[kiae291-B1] Abshire JR , Rowlands CJ, Ganesan SM, So PT, Niles JC. Quantification of labile heme in live malaria parasites using a genetically encoded biosensor. Proc Natl Acad Sci U S A. 2017:114(11):E2068–E2076. 10.1073/pnas.1615195114 28242687 PMC5358388

[kiae291-B2] Bairwa G , Sánchez-León E, Do E, Jung WH, Kronstad JW. A cytoplasmic heme sensor illuminates the impacts of mitochondrial and vacuolar functions and oxidative stress on heme-iron homeostasis in *Cryptococcus neoformans*. mBio 2020:11(4):e00986–e00920. 10.1128/mBio.00986-20 32723917 PMC7387795

[kiae291-B3] Briand VA , Thilakarathne V, Kasi RM, Kumar CV. Novel surface plasmon resonance sensor for the detection of heme at biological levels via highly selective recognition by apo-hemoglobin. Talanta 2012:99:113–118. 10.1016/j.talanta.2012.05.026 22967529

[kiae291-B4] Donegan RK , Moore CM, Hanna DA, Reddi AR. Handling heme: the mechanisms underlying the movement of heme within and between cells. Free Radic Biol Med. 2019:133:88–100. 10.1016/j.freeradbiomed.2018.08.005 30092350 PMC6363905

[kiae291-B5] Espinas NA , Kobayashi K, Sato Y, Mochizuki N, Takahashi K, Tanaka R, Masuda T. Allocation of heme is differentially regulated by ferrochelatase isoforms in Arabidopsis cells. Front Plant Sci. 2016:7:1326. 10.3389/fpls.2016.01326 27630653 PMC5005420

[kiae291-B6] Espinas NA , Kobayashi K, Takahashi S, Mochizuki N, Masuda T. Evaluation of unbound free heme in plant cells by differential acetone extraction. Plant Cell Physiol. 2012:53(7):1344–1354. 10.1093/pcp/pcs067 22555813

[kiae291-B7] Fan T , Roling L, Hedtke B, Grimm B. FC2 stabilizes POR and suppresses ALA formation in the tetrapyrrole biosynthesis pathway. New Phytol. 2023:239(2):624–638. 10.1111/nph.18952 37161708

[kiae291-B8] Fan T , Roling L, Meiers A, Brings L, Ortega-Rodés P, Hedtke B, Grimm B. Complementation studies of the *Arabidopsis fc1* mutant substantiate essential functions of ferrochelatase 1 during embryogenesis and salt stress. Plant Cell Environ. 2019:42(2):618–632. 10.1111/pce.13448 30242849

[kiae291-B9] Grimm B . Primary structure of a key enzyme in plant tetrapyrrole synthesis: glutamate 1-semialdehyde aminotransferase. Proc Natl Acad Sci U S A. 1990:87(11):4169–4173. 10.1073/pnas.87.11.4169 2349227 PMC54069

[kiae291-B10] Hanna DA , Harvey RM, Martinez-Guzman O, Yuan X, Chandrasekharan B, Raju G, Outten FW, Hamza I, Reddi AR. Heme dynamics and trafficking factors revealed by genetically encoded fluorescent heme sensors. Proc Natl Acad Sci U S A. 2016:113(27):7539–7544. 10.1073/pnas.1523802113 27247412 PMC4941510

[kiae291-B11] Hanna DA , Hu R, Kim H, Martinez-Guzman O, Torres MP, Reddi AR. Heme bioavailability and signaling in response to stress in yeast cells. J Biol Chem. 2018:293(32):12378–12393. 10.1074/jbc.RA118.002125 29921585 PMC6093230

[kiae291-B12] Hedtke B , Strätker SM, Pulido ACC, Grimm B. Two isoforms of *Arabidopsis* protoporphyrinogen oxidase localize in different plastidal membranes. Plant Physiol. 2023:192(2):871–885. 10.1093/plphys/kiad107 36806676 PMC10231370

[kiae291-B13] Hoober JK , Kahn A, Ash DE, Gough S, Kannangara CG. Biosynthesis of delta-aminolevulinate in greening barley leaves. IX. Structure of the substrate, mode of gabaculine inhibition, and the catalytic mechanism of glutamate 1-semialdehyde aminotransferase. Carlsberg Res Commun. 1988:53(1):11–25. 10.1007/BF02908411 3256306

[kiae291-B14] Hu X , Gu T, Khan I, Zada A, Jia T. Research progress in the interconversion, turnover and degradation of chlorophyll. Cells 2021:10(11):3134. 10.3390/cells10113134 34831365 PMC8621299

[kiae291-B15] Kim H , Moore CM, Mestre-Fos S, Hanna DA, Williams LD, Reddi AR, Torres MP. Depletion assisted hemin affinity (DAsHA) proteomics reveals an expanded landscape of heme-binding proteins in the human proteome. Metallomics 2023:15(3):mfad004. 10.1093/mtomcs/mfad004 36669767 PMC10022665

[kiae291-B16] Leung GC , Fung SS, Gallio AE, Blore R, Alibhai D, Raven EL, Hudson AJ. Unravelling the mechanisms controlling heme supply and demand. Proc Natl Acad Sci U S A. 2021:118(22):e2104008118. 10.1073/pnas.2104008118 PMC8179208 34035176

[kiae291-B17] Masuda T , Takahashi S. Chemiluminescent-based method for heme determination by reconstitution with horseradish peroxidase apo-enzyme. Anal Biochem. 2006:355(2):307–309. 10.1016/j.ab.2006.04.008 16701068

[kiae291-B18] Richter AS , Banse C, Grimm B. The GluTR-binding protein is the heme-binding factor for feedback control of glutamyl-tRNA reductase. eLife 2019:8:e46300. 10.7554/eLife.46300 31194674 PMC6597238

[kiae291-B19] Richter AS , Nägele T, Grimm B, Kaufmann K, Schroda M, Leister D, Kleine T. Retrograde signaling in plants: a critical review focusing on the GUN pathway and beyond. Plant Commun. 2023:4(1):100511. 10.1016/j.xplc.2022.100511 36575799 PMC9860301

[kiae291-B20] Shimizu T , Yasuda R, Mukai Y, Tanoue R, Shimada T, Imamura S, Tanaka K, Watanabe S, Masuda T. Proteomic analysis of haem-binding protein from *Arabidopsis thaliana* and *Cyanidioschyzon merolae*. Philos Trans R Soc Lond B Biol Sci. 2020:375(1801):20190488. 10.1098/rstb.2019.0488 32362261 PMC7209954

[kiae291-B21] Song Y , Yang M, Wegner SV, Zhao J, Zhu R, Wu Y, He C, Chen PR. A genetically encoded FRET sensor for intracellular heme. ACS Chem Biol. 2015:10(7):1610–1615. 10.1021/cb5009734 25860383

[kiae291-B22] Taira J , Nakashima Y, Yoshihara S, Koga S, Sueda S, Komatsu H, Higashimoto Y, Takahashi T, Tanioka N, Shimizu H, et al Improvement of heme oxygenase-1-based heme sensor for quantifying free heme in biological samples. Anal Biochem. 2015:489:50–52. 10.1016/j.ab.2015.08.004 26278172

[kiae291-B23] Takeda S , Kamiya N, Nagamune T. A novel protein-based heme sensor consisting of green fluorescent protein and apocytochrome b562. Anal Biochem. 2003:317(1):116–119. 10.1016/s0003-2697(03)00096-4 12729608

[kiae291-B100] Tanaka R, Tanaka A. Tetrapyrrole biosynthesis in higher plants. *Ann Rev Plant Biol*. 2007:58:321–346. 10.1146/annurev.arplant.57.032905.105448 17227226

[kiae291-B24] Terry MJ , Smith AG. A model for tetrapyrrole synthesis as the primary mechanism for plastid-to-nucleus signaling during chloroplast biogenesis. Front Plant Sci. 2013:4:14. 10.3389/fpls.2013.00014 23407626 PMC3570980

[kiae291-B25] Thomas J , Weinstein JD. Measurement of heme efflux and heme content in isolated developing chloroplasts. Plant Physiol. 1990:94(3):1414–1423. 10.1104/pp.94.3.1414 16667847 PMC1077392

[kiae291-B26] Wang P , Ji S, Grimm B. Post-translational regulation of metabolic checkpoints in plant tetrapyrrole biosynthesis. J Exp Bot. 2022:73(14):4624–4636. 10.1093/jxb/erac203 35536687 PMC9992760

[kiae291-B27] Weinstein JD , Beale SI. Separate physiological roles and subcellular compartments for two tetrapyrrole biosynthesis pathway in *Euglena gracilis*. J Biol Chem. 1983:258(11):6799–6807. 10.1016/S0021-9258(18)32293-2 6133868

[kiae291-B28] Wilkinson IVL , Bottlinger M, Harraoui E, Sieber Y, A S. Profiling the heme-binding proteomes of bacteria using chemical proteomics. Angew Chem Int Ed Engl. 2023:62(9):e202212111. 10.1002/anie.202212111 36495310

[kiae291-B29] Wißbrock A , Imhof D. A tough nut to crack: intracellular detection and quantification of heme in malaria parasites by a genetically encoded protein sensor. ChemBioChem 2017:18(16):1561–1564. 10.1002/cbic.201700274 28547798

[kiae291-B30] Woodson JD , Perez-Ruiz JM, Chory J. Heme synthesis by plastid ferrochelatase I regulates nuclear gene expression in plants. Curr Biol. 2011:21(10):897–903. 10.1016/j.cub.2011.04.004 21565502 PMC4886857

