# Supplementary material for: A genetically encoded fluorescent heme sensor detects free heme in plants
Source: Plant Physiol. 2024 May 19;196(2):830–41. doi: 10.1093/plphys/kiae291 (PMC11444292; doi:10.1093/plphys/kiae291)
Supplement: kiae291_Supplementary_Data [file kiae291_supplementary_data.zip › kiae291_Supplementary_Data.pdf]

Supplementary Data

Supplementary Figure S1

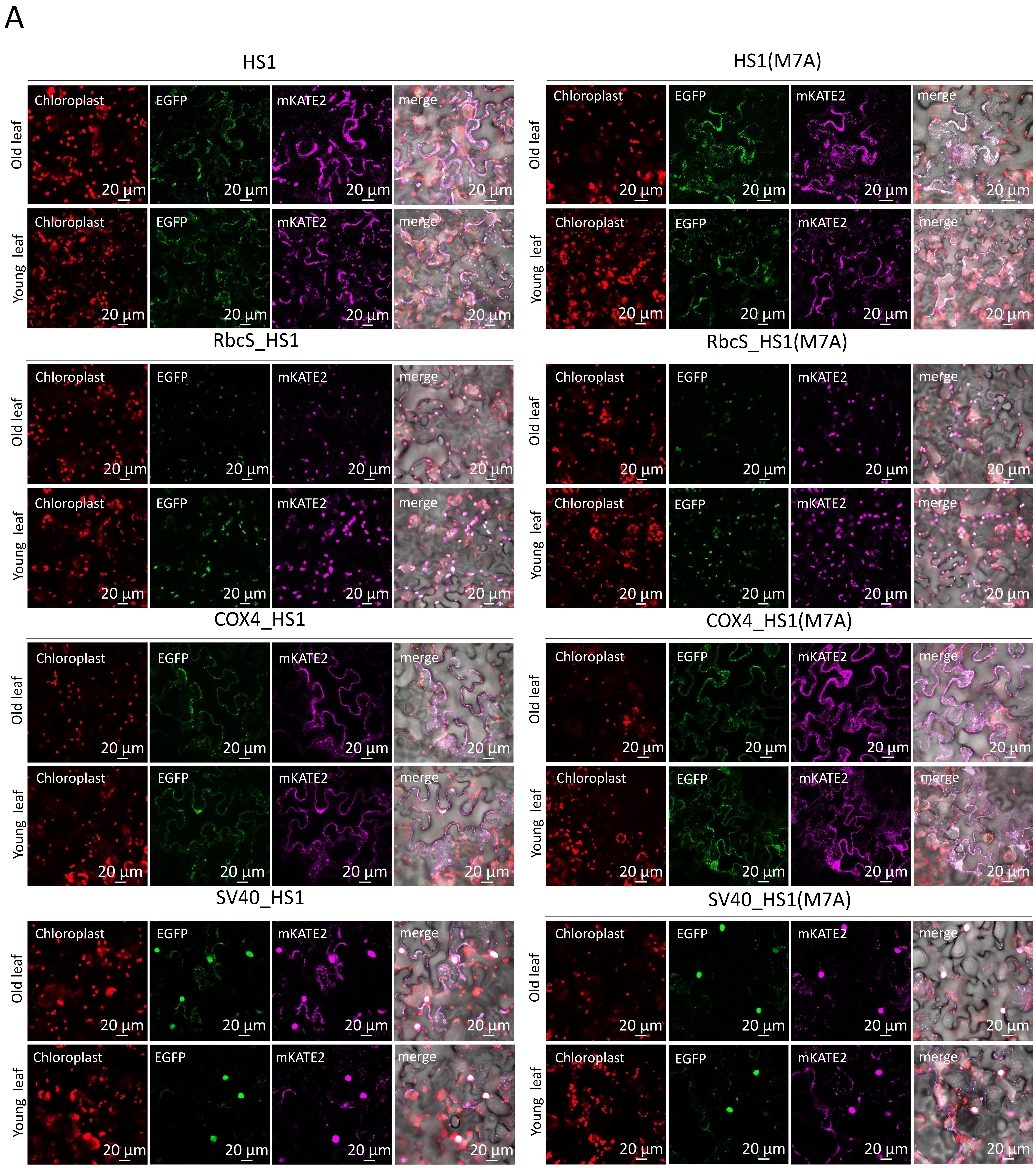

B

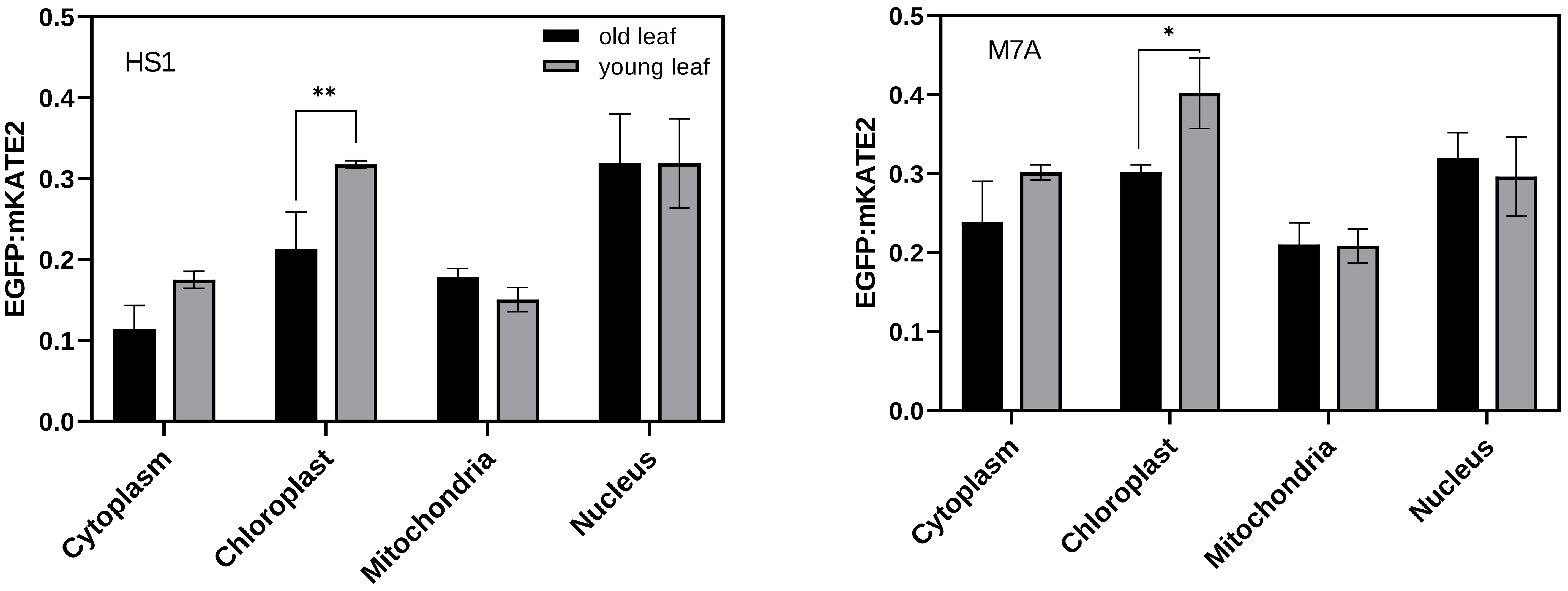

C

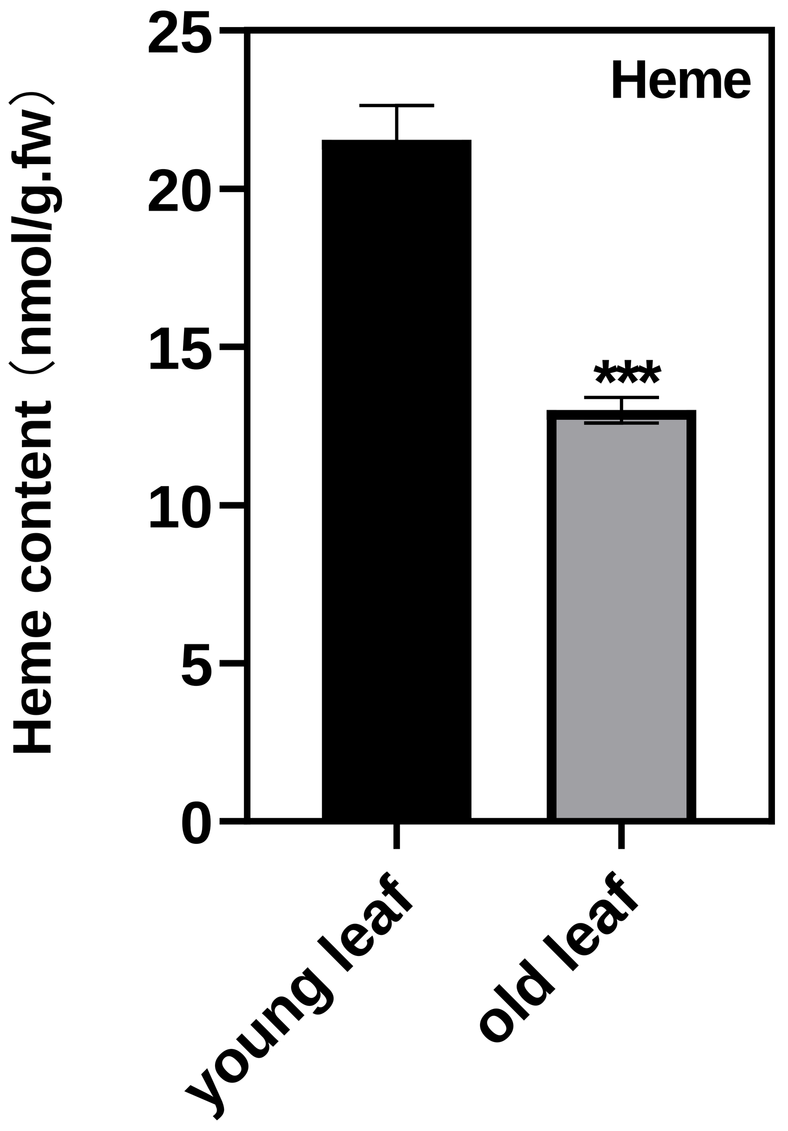

Supplementary Figure S1. Fluorescent signals and EGFP:mKATE2 ratios reported by the heme sensors HS1 and HS1(M7A) following the transient expression of each in old and young leaves of *Nicotiana benthamiana*. A. HS1 and HS1(M7A) fluorescence in the different cellular compartments of transiently transformed old and young tobacco leaves. The images show the fluorescent signal of chlorophyll in chloroplasts, the fluorescent signal emitted by the enhanced green fluorescent protein (EGFP) and the far-red fluorescent protein Katushka 2 (mKATE2) domains in different cellular compartments (cytoplasm, chloroplast, mitochondria, nucleus), and the merged images of the two heme sensors. **B.** EGFP:mKATE2 ratios reported by HS1(M7A) and HS1 in the different cellular compartments of old and young leaves of *Nicotiana benthamiana*. The EGFP:mKATE2 ratio is based on the sum of the pixels in the respective channels. The EGFP:mKATE2 ratios obtained from the EGFP and mKATE2 channels were calculated from five to eight transformed representative cells of different transformants. Note that the ratio is inversely proportional to the level of free heme (see main text). Statistical significance compared with fluorescence in the cytoplasm is indicated by Tukey's HSD method ($P < 0.05$), error bars represent the SD of three biological replicates. **C.** Total heme content in young and old leaf material of *N. benthamiana*. For HPLC analyses, leaves from 6-week-old plants were used. Statistical significance is indicated by the Student's T-test ($P < 0.05$), error bars represent the SD of three biological replicates.

Supplementary Figure S2

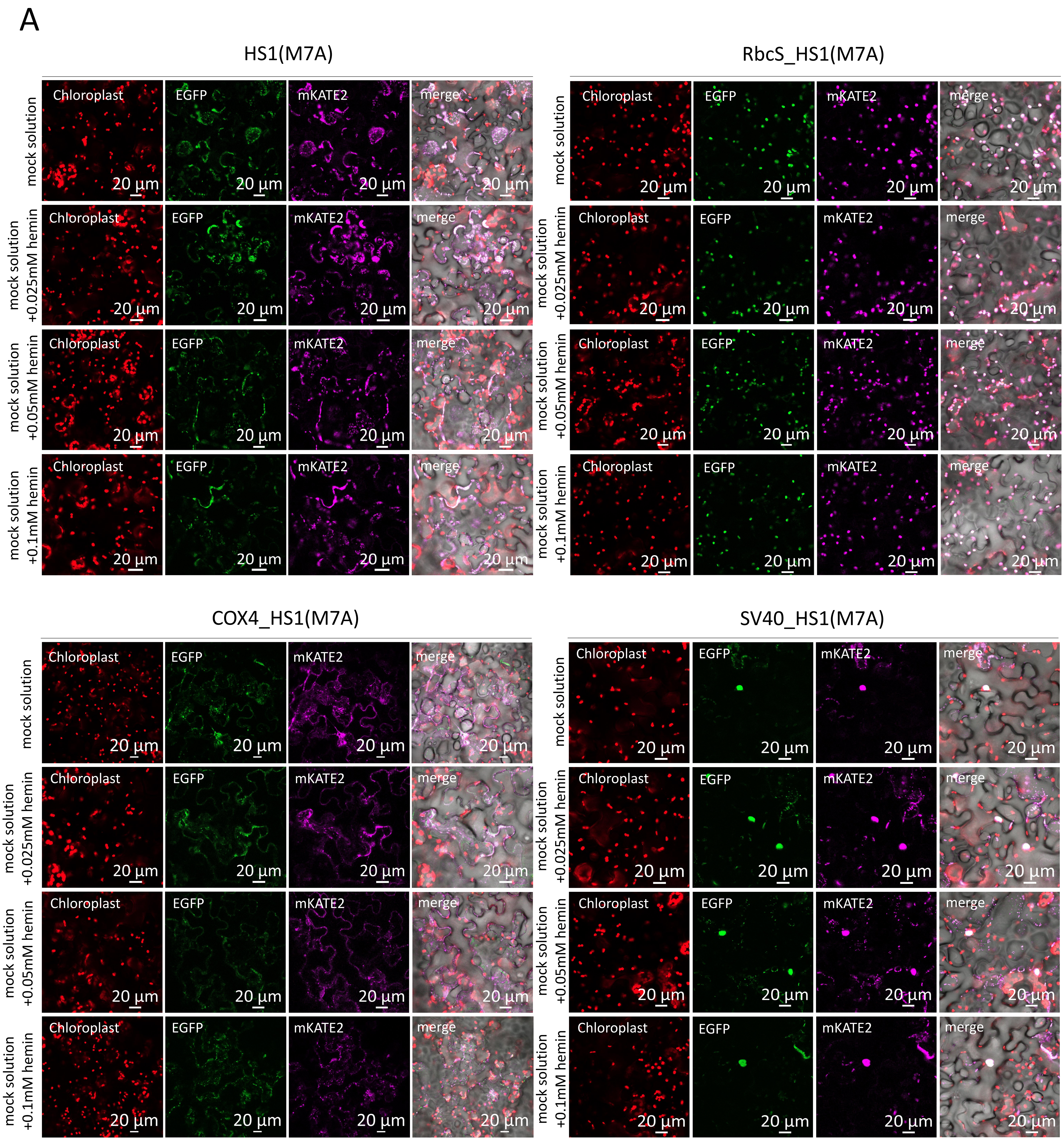

B

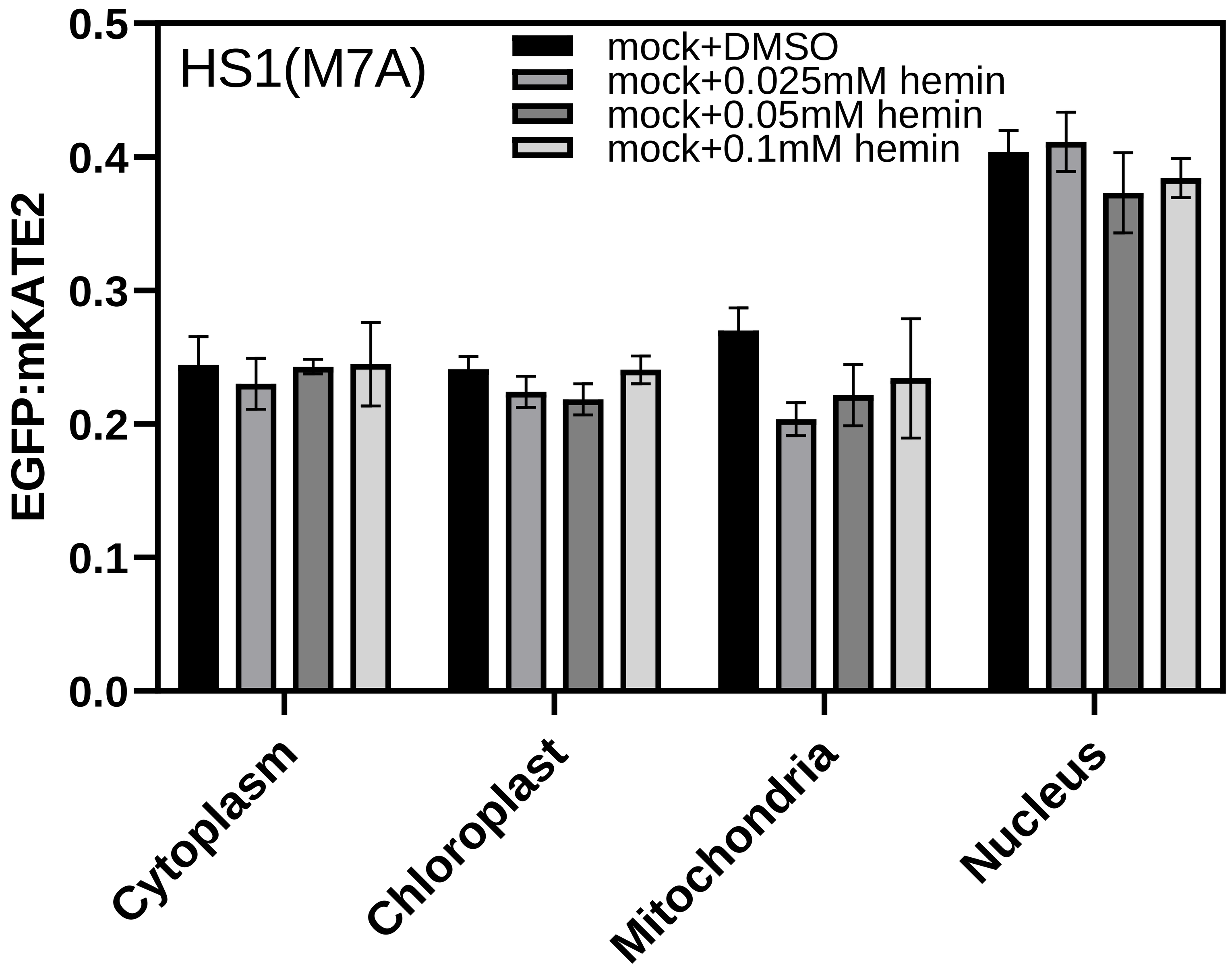

Supplementary Figure S2. Fluorescent signals and EGFP:mKATE2 ratios of HS1(M7A) in different cellular compartments of transiently transformed Nicotiana benthamiana after a treatment with hemin. Transiently transformed tobacco leaves were incubated for 24 hours with different concentrations of hemin (0 mM mock [black] and 0.025mM [grey], 0.05mM [dark grey], 0.1mM [light grey]). **A.** Fluorescent signals of HS1(M7A) in different subcellular compartments of leaf cells of transiently transformed N. benthamiana. Images show the fluorescent signals of chloroplasts, the fluorescent signals of the EGFP domain and mKATE2 domain, respectively in different cellular compartments (cytoplasm, chloroplast, mitochondria, nucleus), and merged images. **B.** Fluorescence ratio EGFP:mKATE2 of HS1(M7A) in the different cellular compartments of N. benthamiana treated with different concentrations of hemin. The fluorescent ratio EGFP:mKATE2 was calculated by the sum of pixels of EGFP and mKATE2 channel.

Supplementary Figure S3

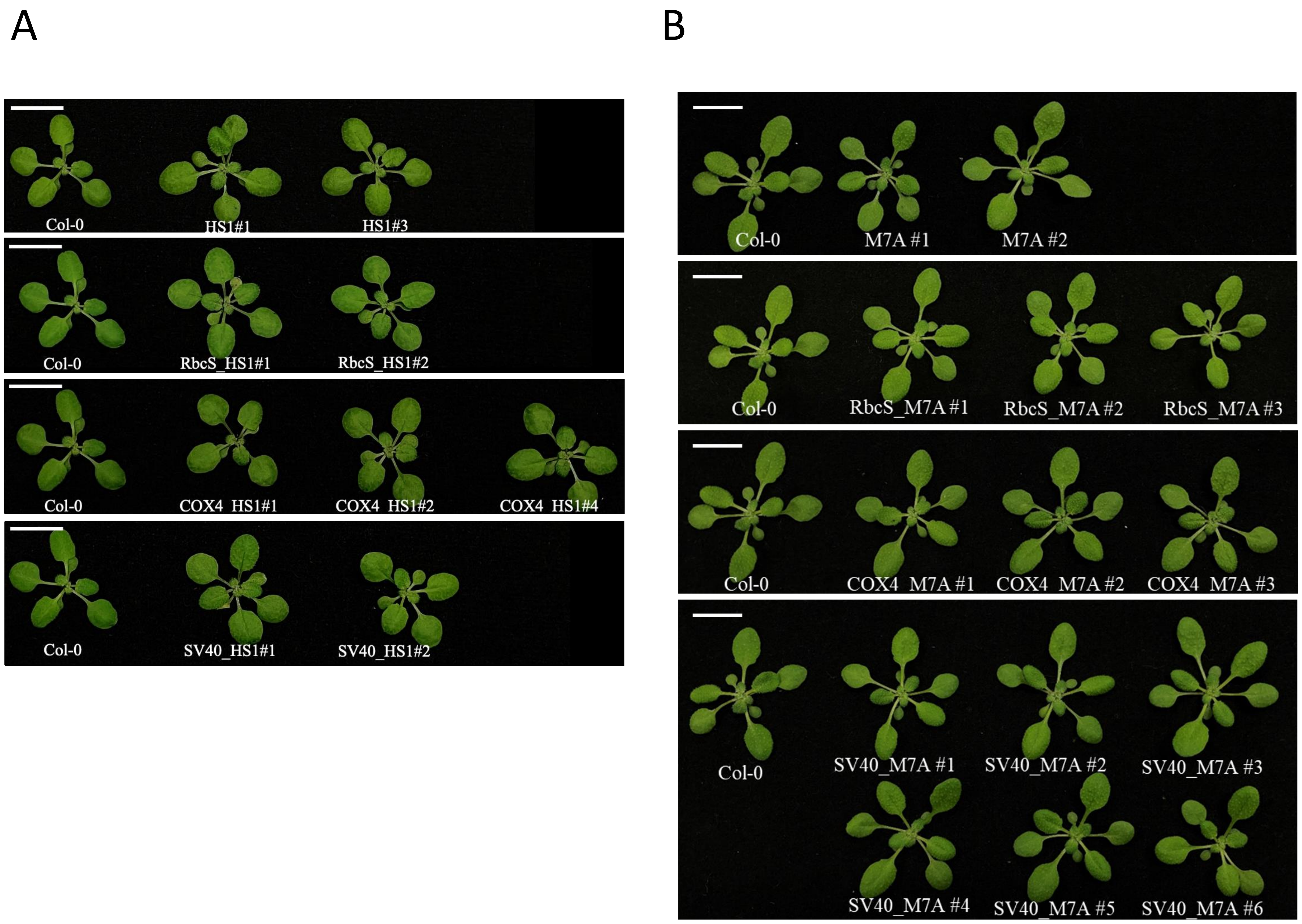

Supplementary Figure S3. Image of *A. thaliana* wild-type and transgenic lines expressing the two heme sensor variants HS1 and HS1(M7A). A. 18-day-old seedlings and, **B.** 20-day-old seedlings are shown which expressed either HS1 and HS1(M7A) in different subcellular compartments. The same Col-0 seedling was displayed within each panel. In dependency to the subcellular localization of cytoplasm, plastids, mitochondria or nucleus the transgenic lines expressing the two heme sensor variants were labelled either HS1#/M7A#, RbcS HS1/RbcS M7A#, COX HS1#/Cox M7A# and SV40 HS1#/SV40 M7A#, respectively. The stably expressing transgenic lines do not phenotypically differ from wild type seedlings. Plants were grown under short-day condition (8 h light/16 h dark) with 100 µmol photons $m^{-2}s^{-1}$ light intensity, 23°C, and 60% relative humidity. Scale bar in each panel

Supplementary Figure S4

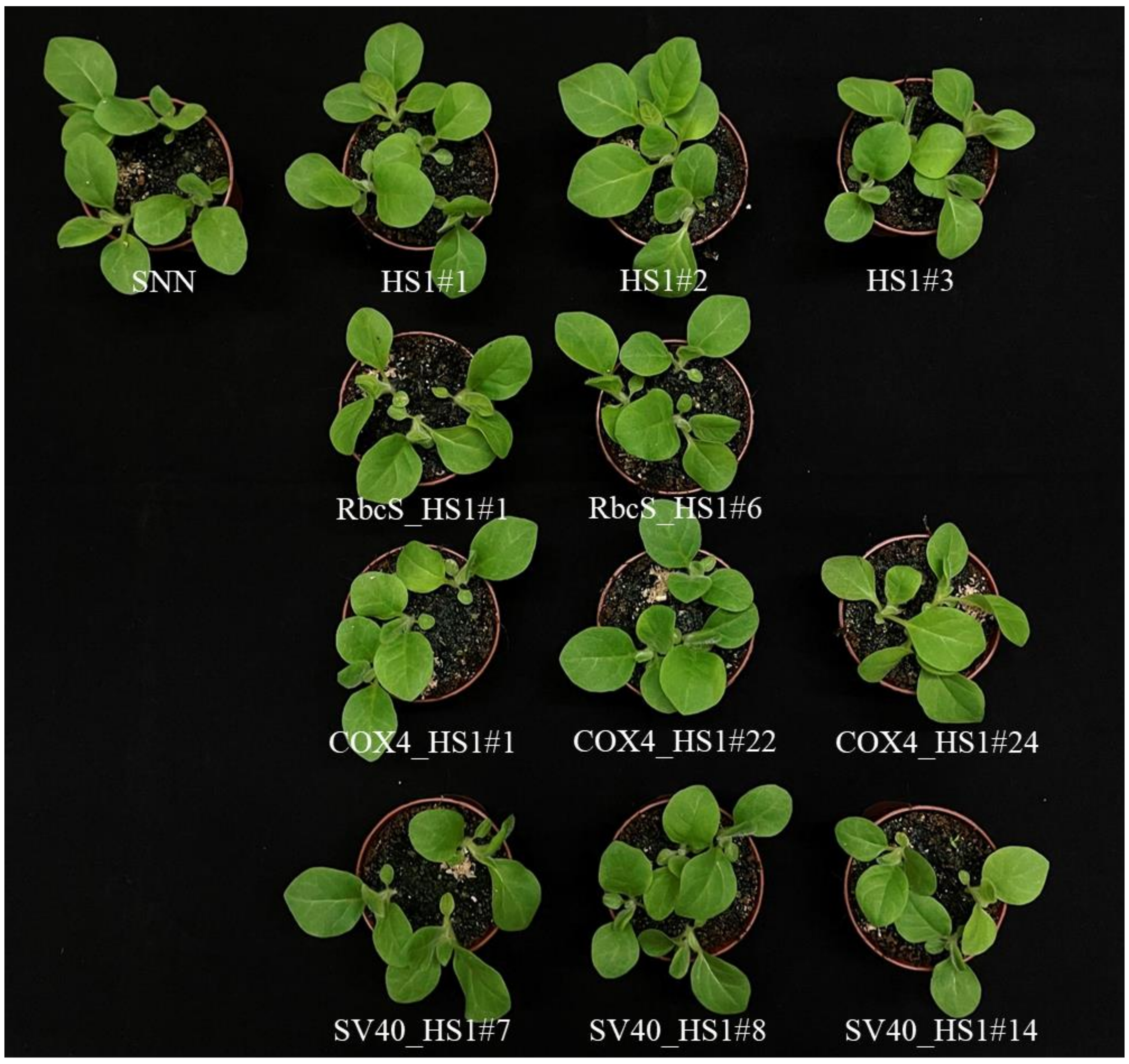

Supplementary Figure S4. Image of 20-day-old *N. tabacum* wild-type and transgenic lines expressing the heme sensor HS1 in different subcellular compartments. In dependency to the subcellular localization of cytoplasm, plastids, mitochondria or nucleus the transgenic lines expressing HS1 were labelled either HS1#, RbcS HS1, COX HS1# and SV40 HS1#, respectively. The stably HS1-expressing transgenic lines do not phenotypically differ from wild type seedlings. Plants were grown under short-day condition (8 h light/16 h dark) with 100 μmol photons $m^{-2}s^{-1}$ light intensity, 23°C, and 60% relative humidity. The plants were grown under continuous light conditions with 100 μmol photons $m^{-2}s^{-1}$ light intensity, 23°C, and 60% relative humidity.

Supplementary Figure S5

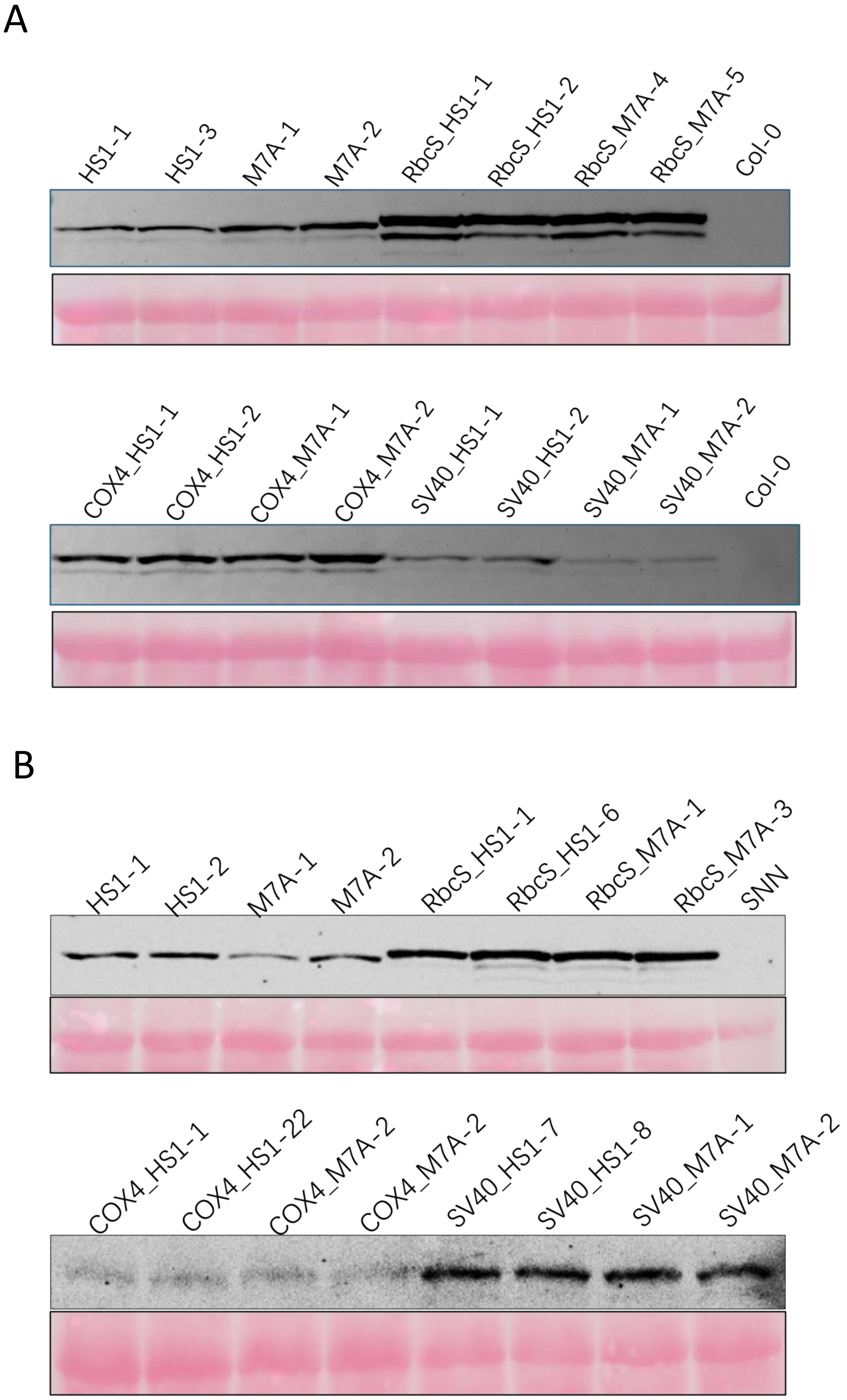

Supplementary Figure S5: Immune analysis of the heme sensor variants HS1 and HS1(M7A) in stably transformed *Arabidopsis thaliana* and *Nicotiana tabacum* lines. Protein extracts from leaves of always two lines expressing one the two heme sensor variants in Arabidopsis (Col-0, **A.**) and tobacco (SNN, **B.**) were analyzed for the content of the heme sensor. The heme sensor variants were expressed either in the cytoplasm (HS1 and M7A), the plastids (RbcS_HS1 and RbcS_M7A), the mitochondria (COX4_HS1 and COX_M7A) and the nucleus (SV40_HS1 and SV40_M7A).

Supplementary Figure S6

A

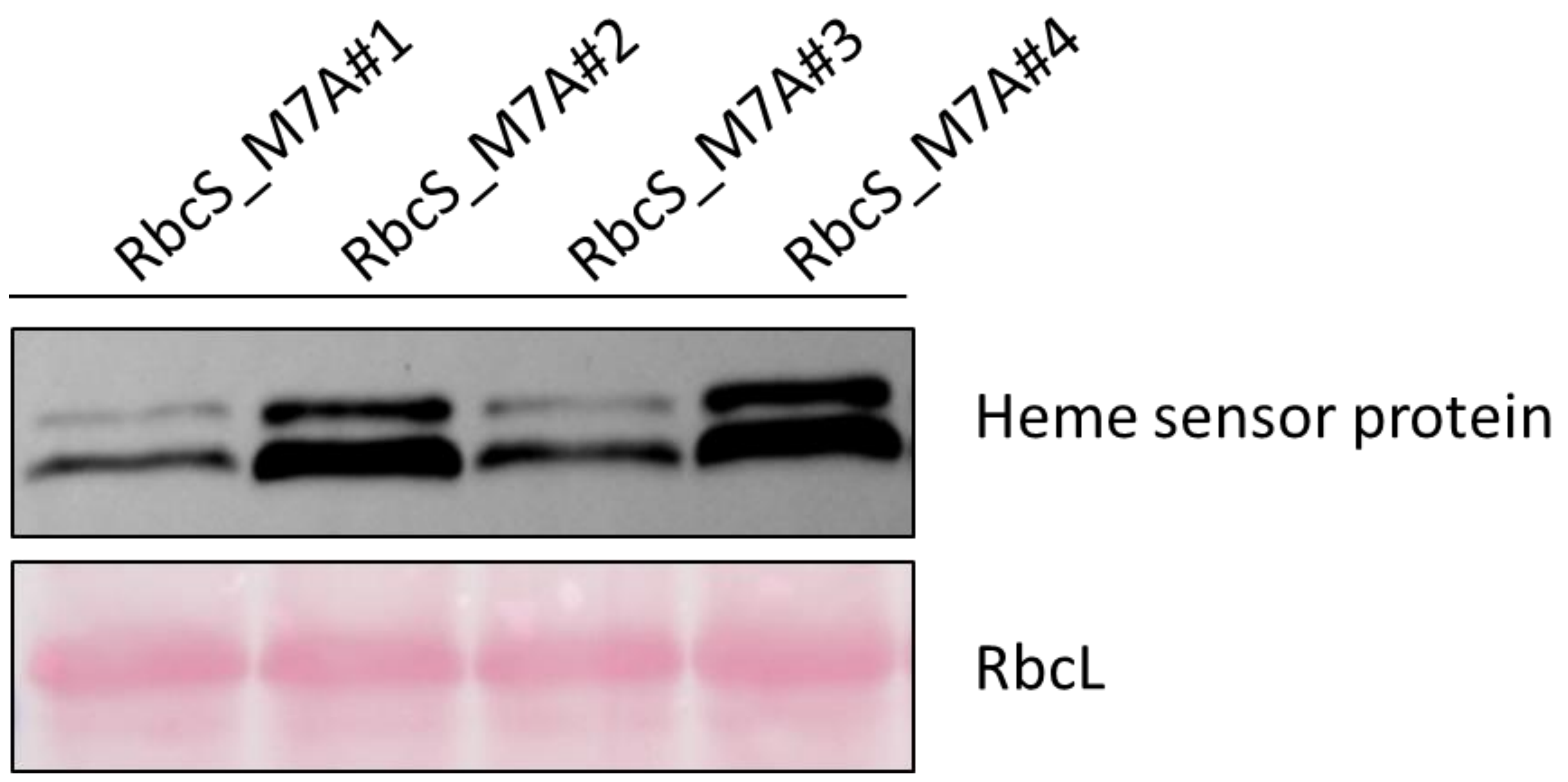

B

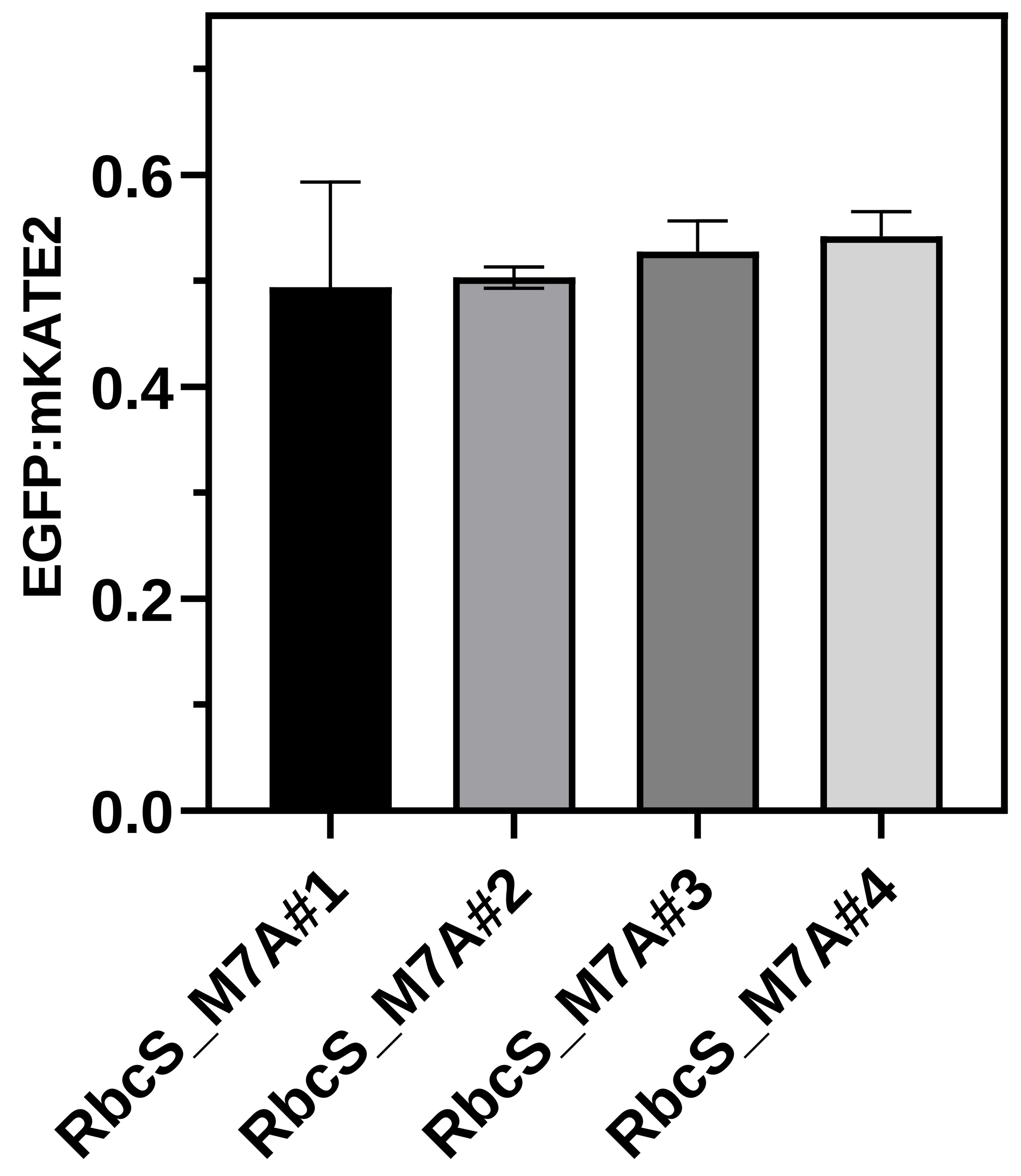

Supplementary Figure S6. Determination of the EGFP:mKATE2 ratio reported by HS1(M7A) in transgenic tobacco lines. *A. thaliana* was stably transformed with the transgene derivative RbcS_HS1(M7A), which enables translocation of HS1(M7A) to chloroplasts**. A.** Immunoblot analysis of the HS1(M7A) sensor levels detected in four different, stably transformed RbcS_HS1(M7A) Arabidopsis mutant lines. The GFP antibody was used to determine the amount of the HS1(M7A) protein. **B.** EGFP:mKATE2 ratios detected expressed RbcS_HS1(M7A) in 4 stably transformed Arabidopsis lines. The EGFP:mKATE2 fluorescent ratio is is derived from the the numbers of pixels in the EGFP and mKATE2 channels. For each line at least three different progenies of the T3 generation were analyzed. The EGFP:mKATE2 ratios obtained from the EGFP and mKATE2 channels were calculated from five to eight transformed representative cells of different transformants. Note that the ratio is inversely proportional to the level of free heme (see main text). Statistical significance compared with fluorescence in the cytoplasm is indicated by Tukey's HSD method (P < 0.05), error bars represent the SD of three biological replicates.

Supplementary Figure S7

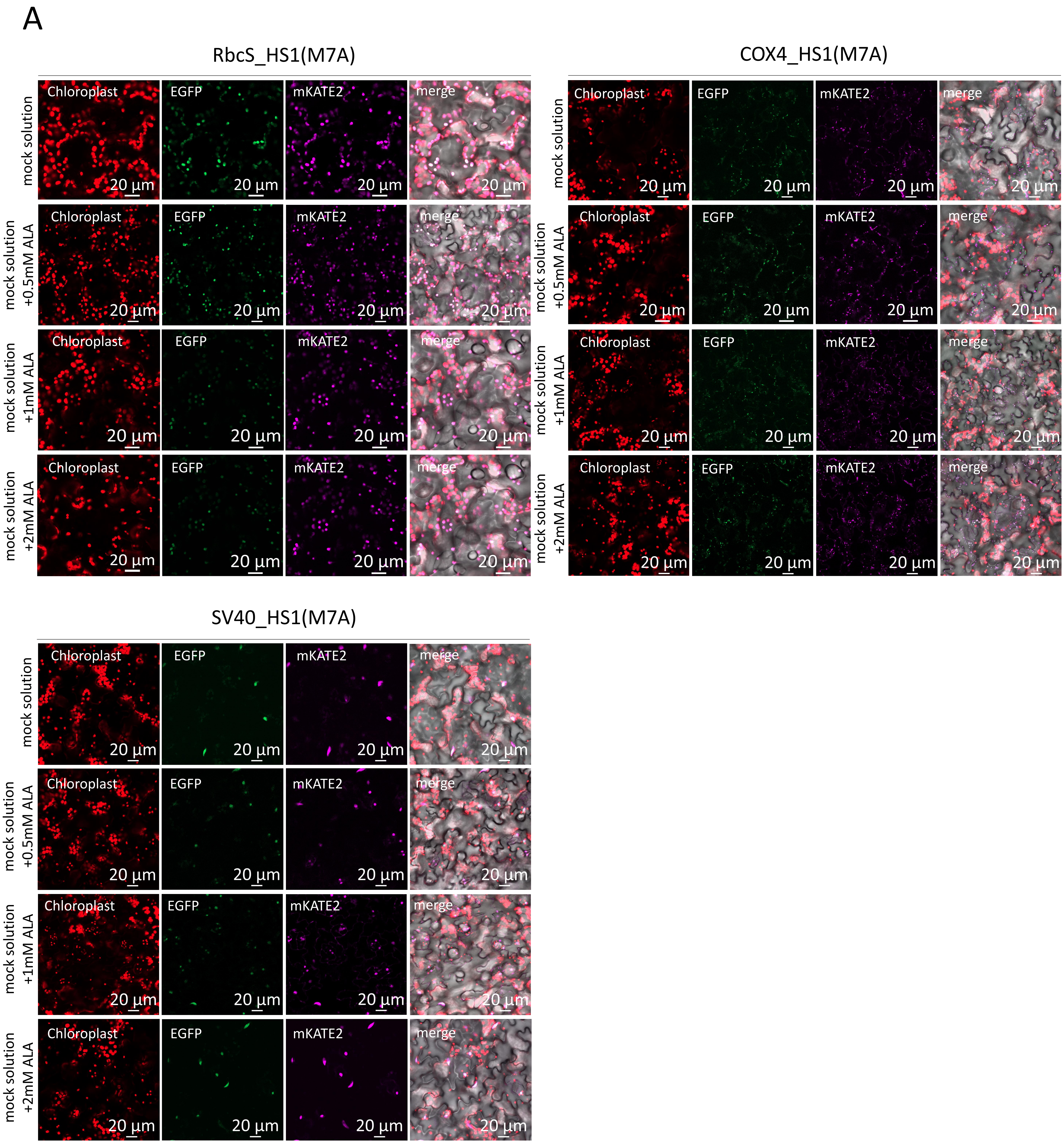

B

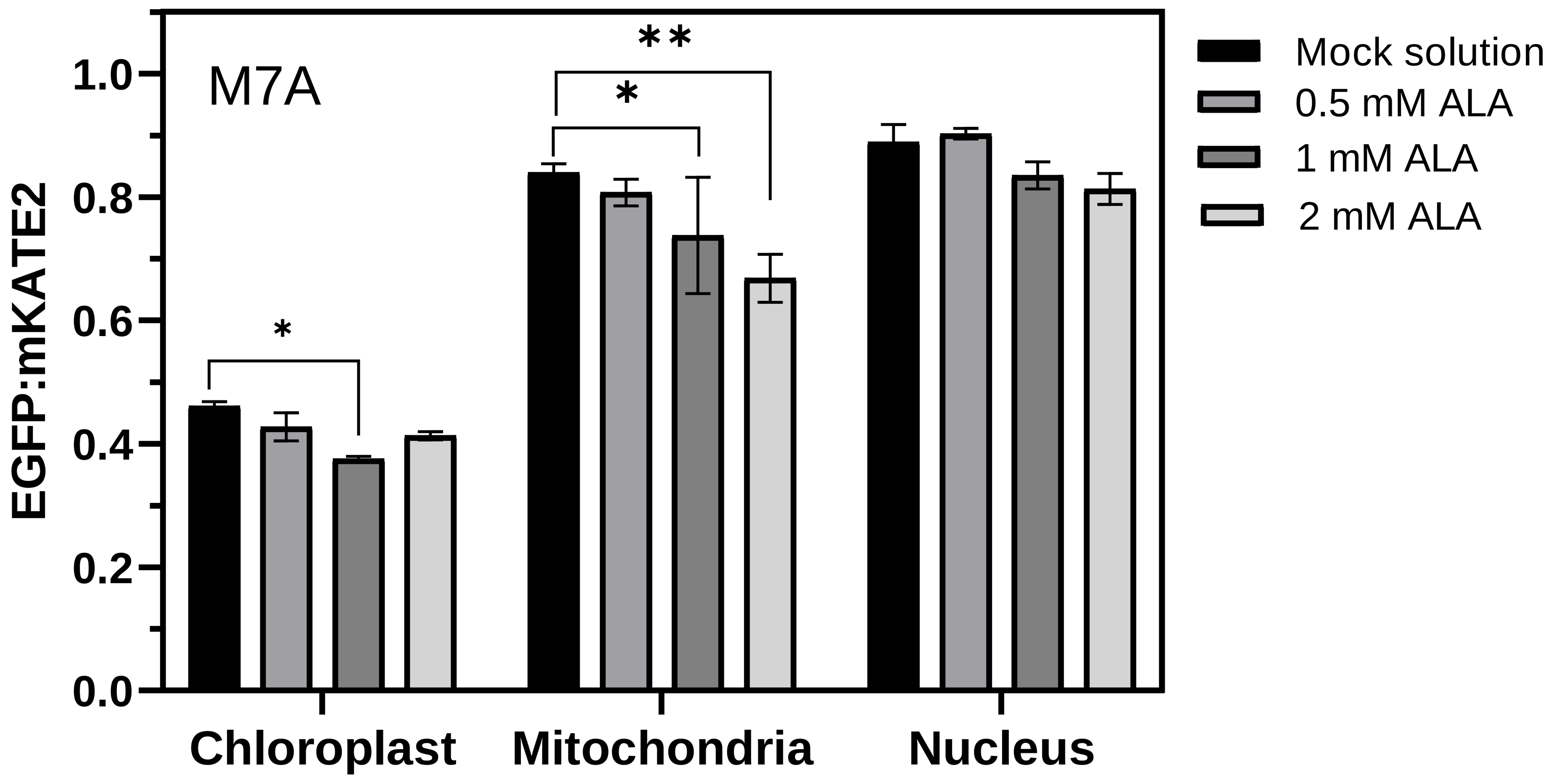

C

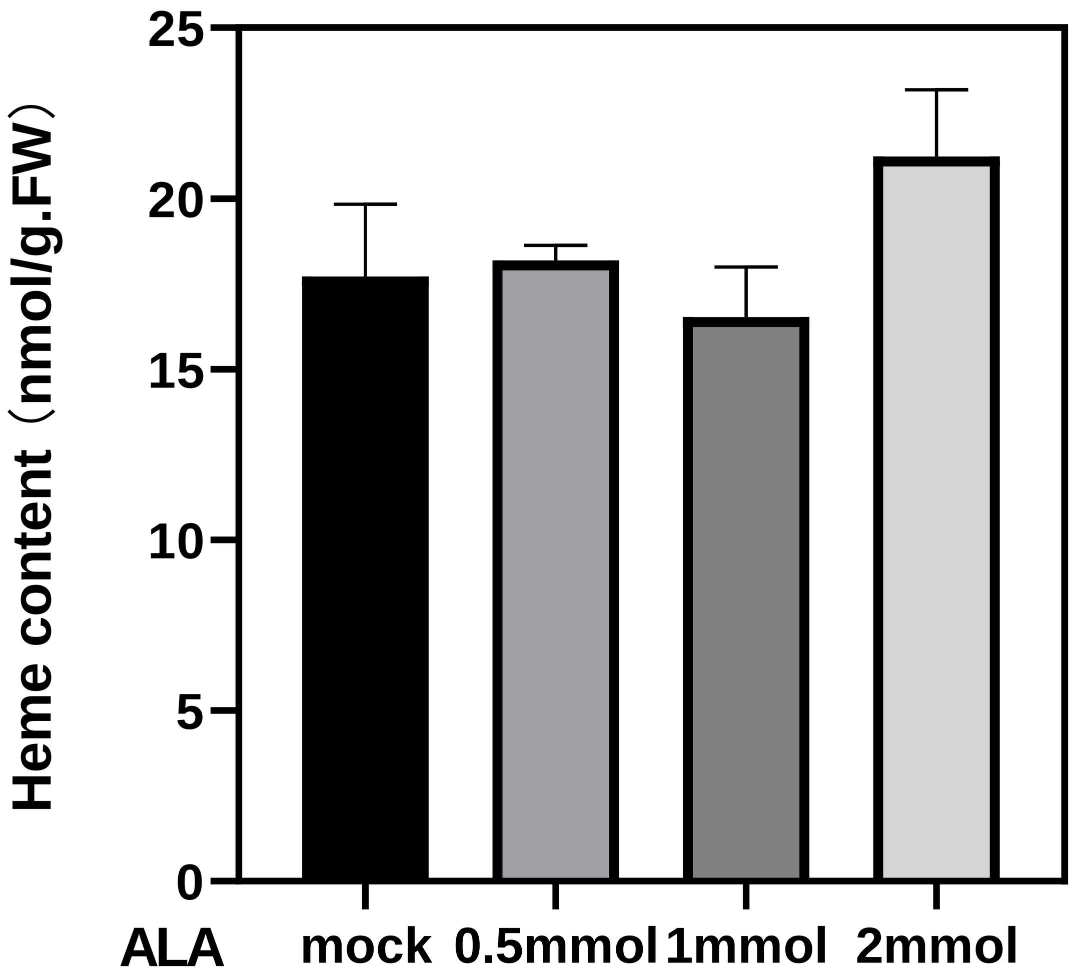

Supplementary Figure S7. Fluorescence signals of the heme sensor protein and ratio of the EGFP:mKATE2 fluorecence signal of three different HS1(M7A) stably transformed 4-week-old Arabidopsis lines after treatment with 5-aminolevulinic acid (ALA) for 24 h. The transgenic HS1(M7A) was translocated into chloroplasts, mitochondria and nuclei. Leaf disks were incubated with different concentrations (0 mM, 0.5 mM, 1 mM, 2 mM) of ALA solution. **A.** Fluorescence signals reported by HS1(M7A) in the Arabidopsis lines after feeding the leaves with different concentrations of ALA. The images show the chlorophyll fluorescence in the chloroplasts, the EGFP and mKATE2 signals detected by HS1(M7A) in the various cellular compartments (chloroplast, mitochondria, nucleus), and the merged image of the fluoresence of the two fluorescence protein domains. **B. E**GFP:mKATE2 ratio reported by HS1(M7A) in the transgenic Arabidopsis lines after incubation with different concentrations of ALA. The EGFP:mKATE2 ratio is based on the numbers of pixels in the respective channels. **C.** Non-covalently bound heme content of leaves of the different transgenic Arabidopsis lines after the ALA treatment of their leaves. The heme content was determined by HPLC analyses.

Supplementary Figure S8

A

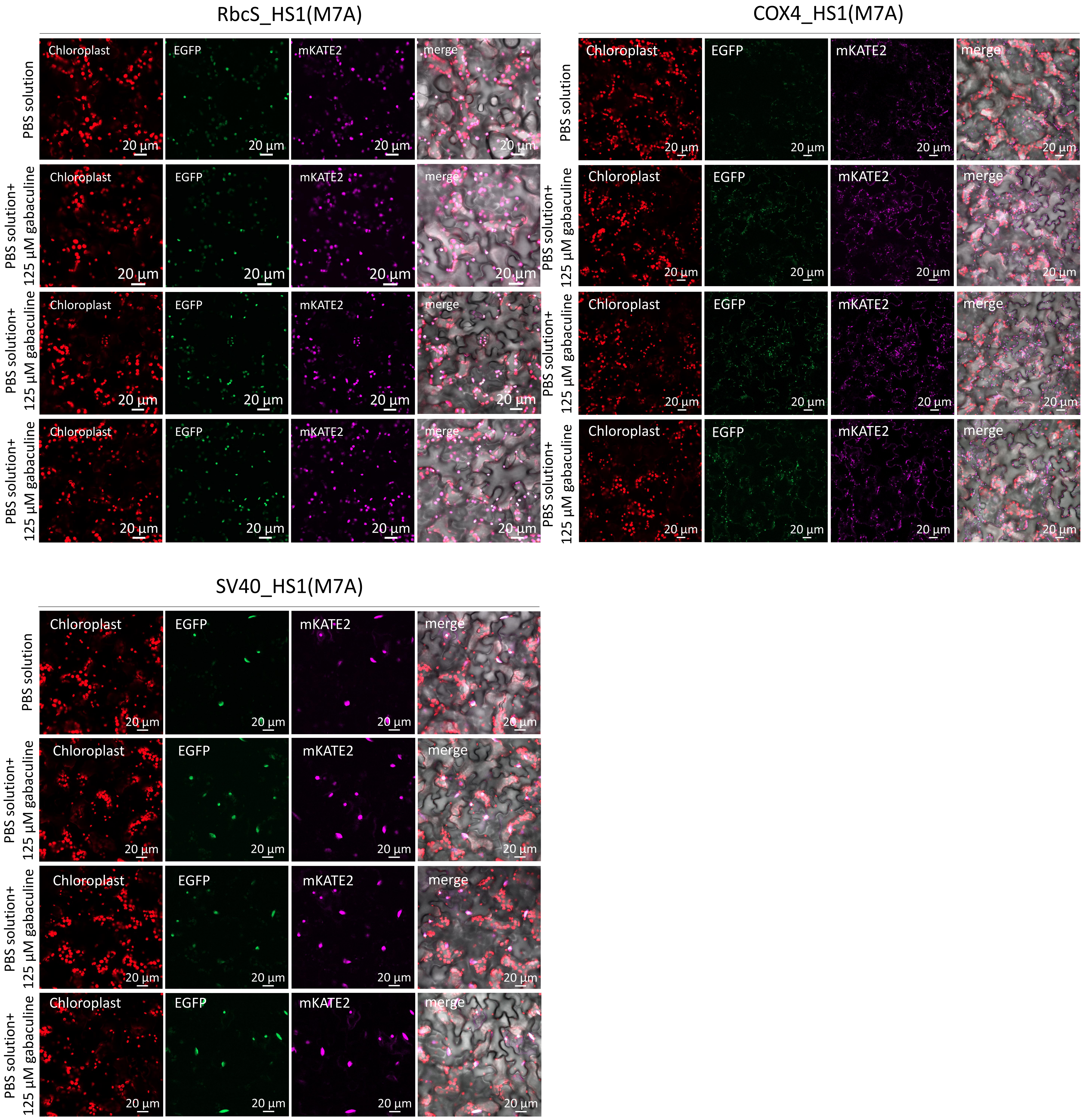

B

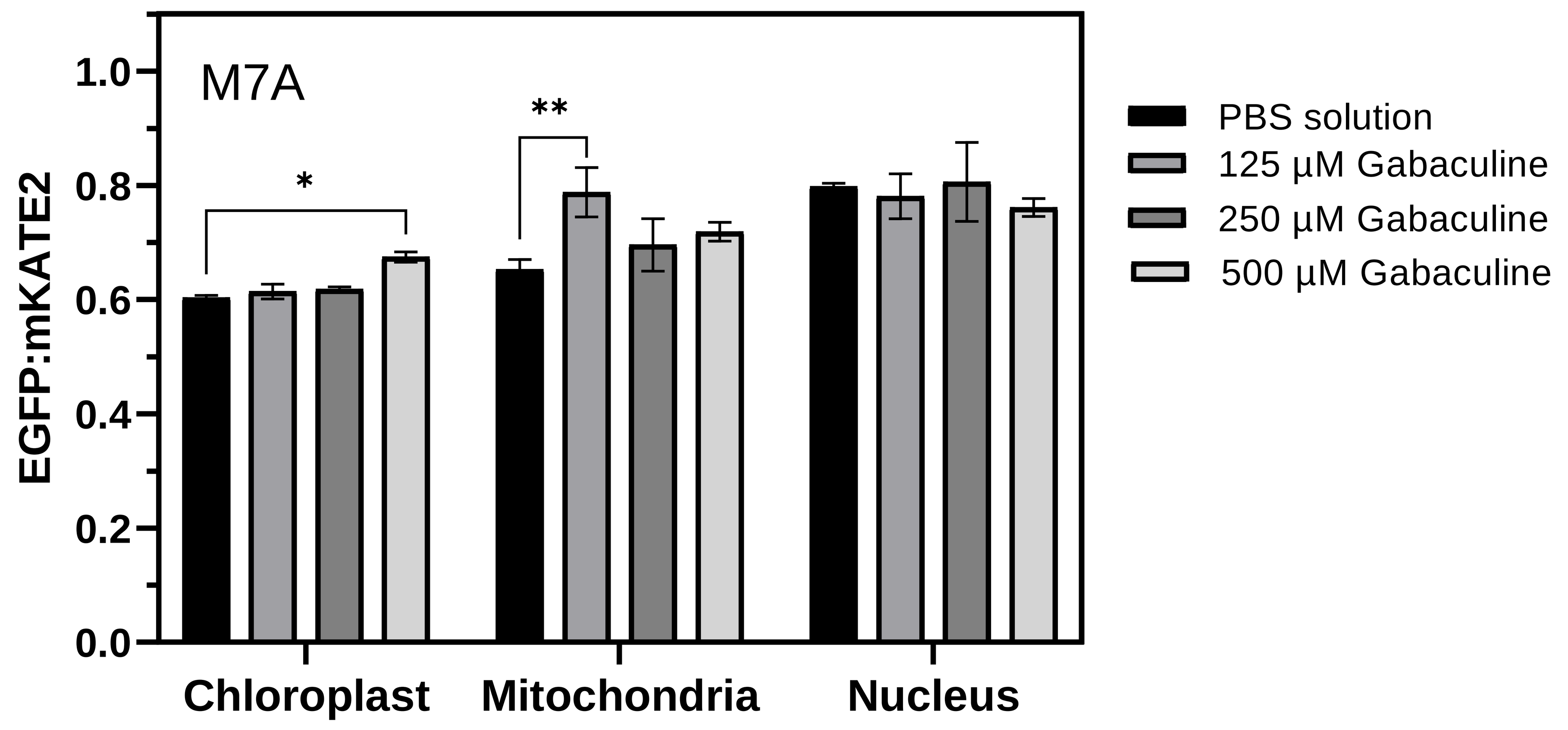

C

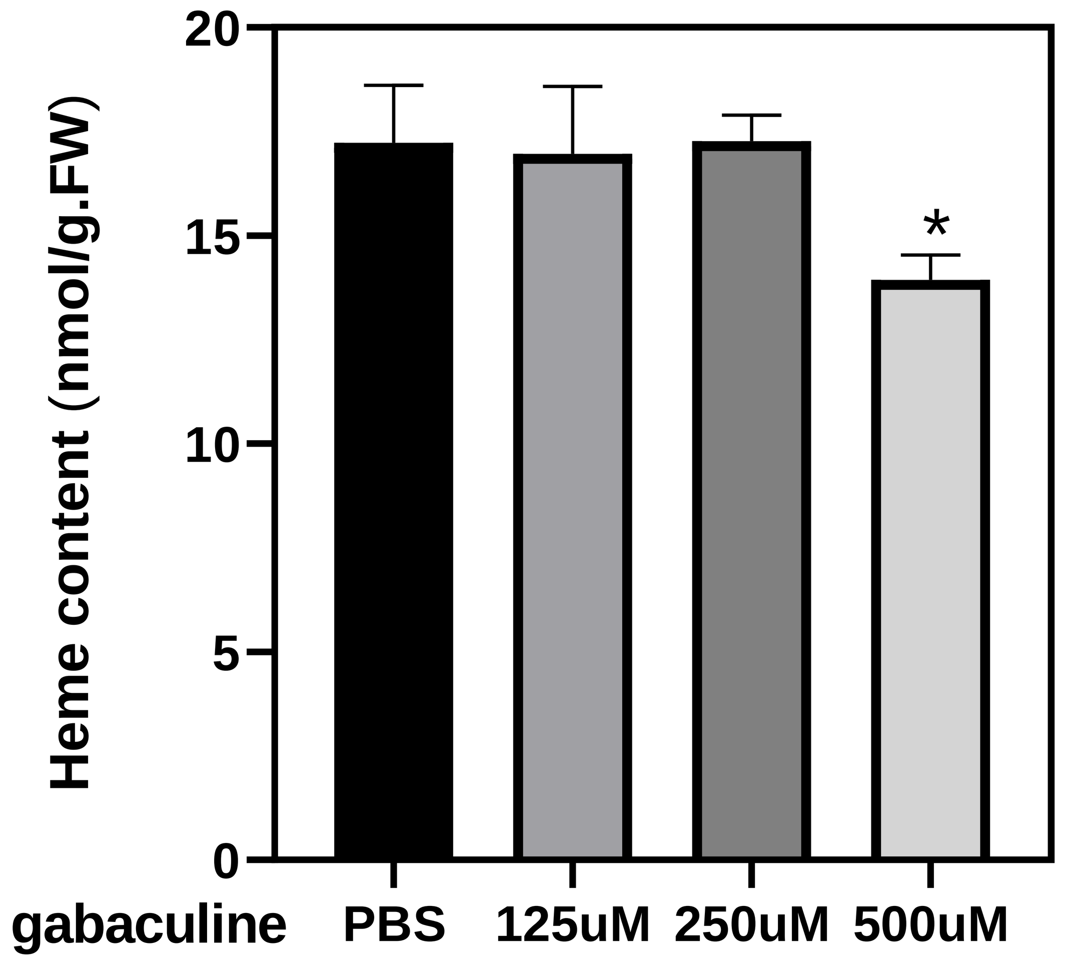

Supplementary Figure S8. EGFP:mKATE2 ratios reported for three different stably transformed 4-week-old Arabidopsis lines that had been treated with the TBP inhibitor gabaculine for 24 h. The HS1(M7A) sensor was translocated into chloroplasts, mitochondria and nuclei. Leaf disks were incubated with different concentrations (0 mM, 125 µM, 250 µM, 500 µM) of gabaculine solution. **A**. Fluorescence signal reported by HS1(M7A) in the Arabidopsis lines after treatment of their leaves with different concentrations of gabuculine. The images show the chlorophyll signal in the chloroplasts, the EGFP and mKATE2 signals in the different cellular compartments (chloroplast, mitochondria, nucleus), and the merged EGFP/mKATE2 image. **B.** EGFP:mKATE2 of HS1(M7A) in the transgenic Arabidopsis lines expressing HS1(M7A) after incubation with different concentrations of gabaculine. The fluorescence signal ratio of EGFP and mKATE2 is calculated by the sum of pixels of EGFP and mKATE2 channel. **C**. Non-covalently bound heme content of leaves of the different transgenic Arabidopsis lines following gabaculine treatment. The heme content was determined by HPLC analyses.

Supplementary Figure S9

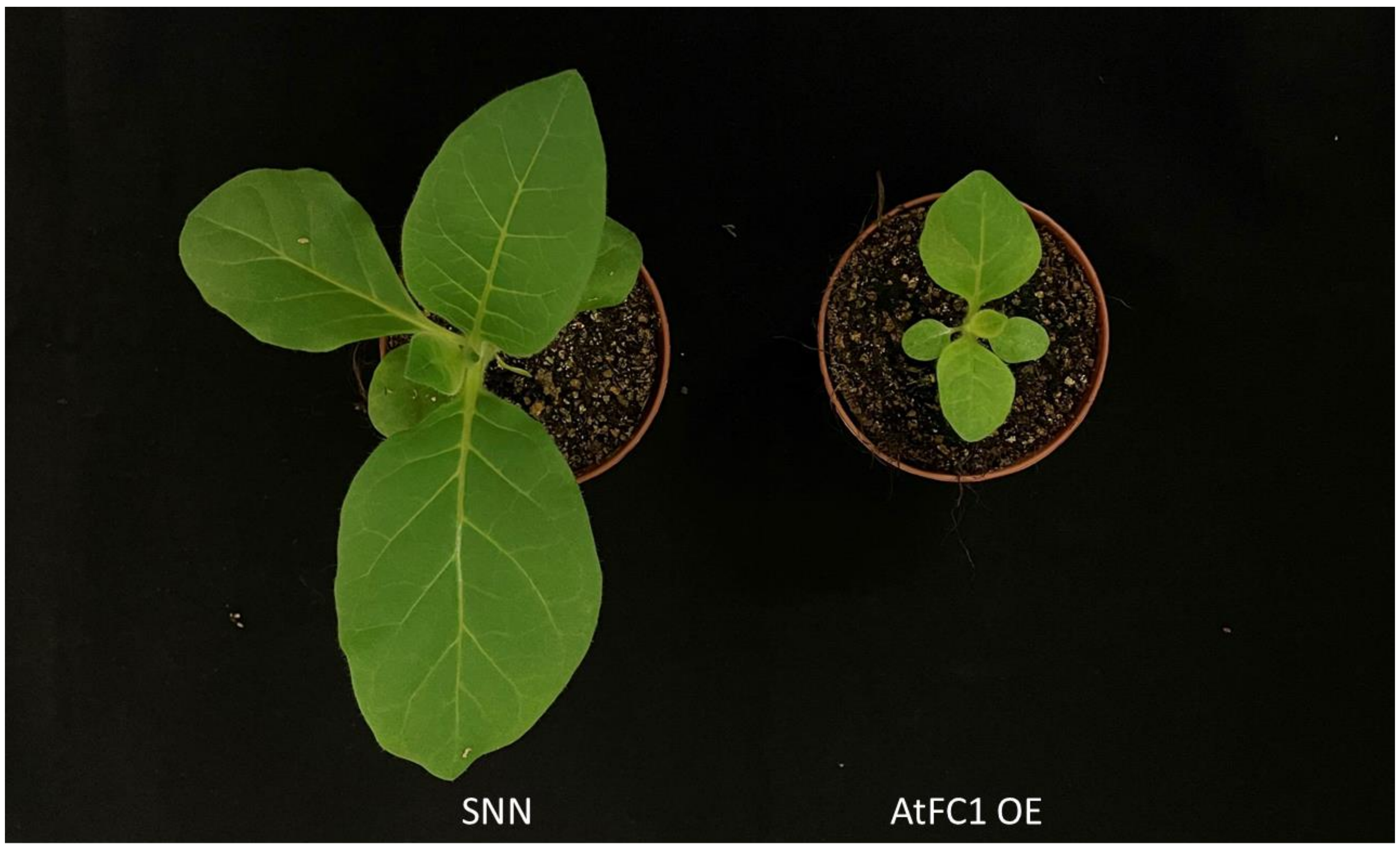

Supplementary Figure S9. Image of seedlings of a representative *AtFC1* overexpression line and wild type of *N. tabacum*. The plants grew 34 days under continuous light conditions at 100 µmol photons $m^{-2}s^{-1}$ light, 23°C, and 60% relative humidity. The AtFC1 expressing tobacco line (AtFC1 OE) is characterized by a slightly yellow-green leaf pigmentation (Fan et al., 2019; Fan T, Roling L, Meiers A, Brings L, Ortega-Rodés P, Hedtke B, Grimm B. Complementation studies of the Arabidopsis fc1 mutant substantiate essential functions of ferrochelatase 1 during embryogenesis and salt stress. Plant Cell Environ. 2019 Feb;42(2):618-632. doi: 10.1111/pce.13448).

Supplementary Table S1. Primers used for the subcloning of the gene constructs encoding either the Heme Sensor 1 (HS1) or the variant HS1(M7A).

primer name	sequence
HS1_fwd	ATCTAGAAAAATGCACATGGTATCGGAACT
HS1_rev	AGAGCTCATCATTTATACAGTTCATCCATA
RbcS_HS1 overlap_fwd	TTACCGATTCCCACATGGTATCGGAACT
RbcS_fwd	ATCTAGAAAAATGGCTTCCTCTATGCTCTC
RbcS_HS1 overlap_rev	ATACCATGTGGGAATCGGTAAGGTCA
Cox4_HS1 overlap_fwd	CTGCTTCAGCAACACATGGTATCGGAACTG
COX4_fwd	ATCTAGAAAAATGGTTTCACTACGTCAATC
Cox4_HS1 overlap_rev	TACCATGTGTTGCTGAAGCAGATATCT
SV40_HS1_rev	AGAGCTCATCAAACCTTCCTCTTCTTCT
