# Supplementary material for: A genetically encoded fluorescent heme sensor detects free heme in plants
Source: Plant Physiol. 2024 May 19;196(2):830–41. doi: 10.1093/plphys/kiae291 (PMC11444292; doi:10.1093/plphys/kiae291)
Supplement: kiae291_Supplementary_Data [file pp.00454.2024-s04.pdf › pp.00454.2024-s04.pdf]

Plant Physiology®

Application of a Genetically Encoded Fluorescent Heme Sensor to the Detection of Free Heme in Plants

Bingxiao Wen and Bernhard Grimm

Review Timeline:

Submission Date:	20-Dec-2023
Editorial Decision:	17-Jan-2024
Resubmission Received:	27-Mar-2024
Accepted:	19-Apr-2024

14-Jan-2024

Prof. Bernhard Grimm
Humboldt-Universität zu Berlin
Berlin 10115
Germany

RE: Application of a Genetically Encoded Fluorescent Heme Sensor to the Detection of Free Heme in Plants

Dear Prof. Grimm:

Thank you for giving us the opportunity to review your manuscript for publication in the Plant Physiology. The reviewers have found the manuscript of interest, but have raised a number of concerns that will require your attention. As these concerns will entail substantial changes to the manuscript, you are likely to need additional time to address them. I must therefore reject the manuscript in its present form. Nonetheless, if you are able to address the points raised in review, I would welcome your resubmission of a manuscript that incorporates your responses.

In particular, I direct your attention to the following comments for consideration in a resubmitted manuscript.

Reviewer 1 is requesting phenotypic data of stable transformants and Reviewer 2 for FC1-OE lines. Reviewer 2 is also asking for data from multiple transgenic lines for each construct shown in Figs. 4 and 5, as done for the different Rbcs_HS1(M7A) lines (Arabidopsis or tobacco?) in Supplemental Fig. S3. Further, please provide the information on statistical tests.

Please note that any resubmission will be treated as a new entry to the system and is likely to go out for review.

If you do have any queries or concerns, you are welcome to contact me in the first instance.

Thank you again for allowing Plant Physiology to review your work. We look forward to hearing from you soon.

Sincerely,

Plant Physiology

-- Reviewer comments:
Reviewer #1 (Comments for the Author):

The authors of this manuscript describe elegantly the use of genetically encoded HS1 and HS1 (M7A) heme sensors for monitoring the distribution of free hemes in different plant cellular compartments of Arabidopsis and tobacco plants. As a proof-of-concept they used a smart strategy which combined (i) biochemical and (ii) genetic approaches to manipulate heme content by altering heme metabolism. This opens interesting new possibilities in the field of plant biology for deciphering key mechanisms in heme metabolisms, subcellular allocation, (re)distribution, dynamics, trafficking and heme-dependent signaling. Those yet numerous open questions are of high importance. Indeed, a tightly controlled labile "free" heme content is necessary for fast mobilization for heme-dependent processes while its over-accumulation should be prevented due to its toxicity potential. Despite its importance, the mechanisms or the factors involved in the control of heme distribution remains to date largely unknown.
The use of genetically encoded sensors appears to be a unique strategy in comparison to disruptive enzyme-based assays. It allows high spatial resolution with the targeting of sensors to subcellular compartment and high temporal resolution to quantify steady-state heme levels in situ which would enable accurate visualization of distribution, mobilization and trafficking of heme. This approach is particularly important as no relevant method is yet available for quantifying heme content distribution at subcellular level and heme trafficking from the site of synthesis into the cytoplasm and other subcellular compartment.
Here, the authors adapt the sensors HS1 and HS1(M7A) from Hanna et al. 2016 for its use in plants. They have designed different constructs for their targeting to four subcellular compartments in plants (i.e., cytoplasm, plastids, mitochondria, chloroplast and nucleus). They have first hypothesized that the use of sensors such as HS1 and HS1(M7A) could enable to monitor heme content with high spatial and temporal resolutions in planta and then, have demonstrated experimentally the relevance of such an approach by using genetic, biochemical, and environmental perturbations that intend to manipulate heme contents. Their analyses demonstrate that the use of stably and transiently transformed Arabidopsis and tobacco plants expressing HS1 and HS1(M7A) enables to quantify the relative amounts of freely accessible intracellular heme and detect dynamic changes in its subcellular content. They have shown that associated affinity and expression of HS1 and HS1(M7A) are consistent with a proper function of the sensor in Arabidopsis and tobacco plants.
The importance of such a methodology is high in regards of the importance of heme-dependent proteins. Its relevance for a

broad range of readers from the plant biology field is high. Especially, as few quantitative analyses have been undertaken for the relative amounts of these proteins in different subcellular compartment. A reliable method is necessary for assessing heme bioavailability expected to be tightly regulated temporally and spatially depending on the demands based on its synthesis and allocation for heme-dependent processes. The interest, originality is high while the work is highly focused. The text is clear, easy to read and presented with logic. The conception of the study as its design is relevant.
Finally, the authors provide evidences, arguments and data that support overall the conclusions while it is necessary to provide data supporting the fact that sensor expression do not affect plant developments and growth in stably transformed lines. Ine general, the conclusions respond very well to the main question. There is overall no ambiguity in the interpretations. The study will support effort aiming to decipher distribution, allocation and trafficking of free hemes in plants.
Major problem:
- In line 225, the authors stated that stably transformed Arabidopsis and tobacco lines did not show any aberrations of developments, growth rate, green pigmentation or leaf size. Sensors are considered at best lowly invasive... It is necessary to provide enough evidences/data that there is no significant alteration in plant developments and growth in derivate transgenic lines to validate the relevance of their use in vivo. Please provide data for it.
Minor problems:
- HS1 and HS1(M7A) are named high and medium/lower affinity sensors by the authors. It would be good to remind their affinity somewhere in the text as high or medium/lower affinity is rather subjective.
- Line 7: ORCID.
- Fig.1: The immunoblot shows a very thin signal for SV40_HS1 and SV40_HS1(M7A), please could the authors comment on this? At the opposite, Rbcs_HS1 and Rbcs_HS1(M7A) show both relatively high signals with, apparently, multiple bands. Could the authors comment on this?
- Fig.1-2: The use of ratiometric data enables to get rid off potential effects from variable expression level inherent to sensor expression in vivo. Yet, it is important to provide a view of those differences between the different conditions/constructs. Could the authors put the mKate2 levels in supplementary as proxy for expression levels of the different conditions/constructs?
- Fig. 1: "The EGFP: mKATE2 obtained from the GFP and mKATE2 channels was calculated from more than three transformed representative cells of different transformants." Please provide the exact numbers.
- Line 131: ALA should be defined at first mention.
- Fig. 2B: Y-axis: correct bold bracket symbols.
- Line 147: EGFP:mKATE2
- In line 156: the authors claimed that it is assumed that a rise in the heme pool in the cytoplasm is a results of altered heme metabolism in plastids. I think it is over-concluded. Please argue further or delete this statement.
- In Line 159: I am not convinced by this statement. Please argue further or delete this statement.
- Fig. 3B: Y-axis: correct bold bracket symbols.
- Fig. 4A: Could the authors comment on the multiple bands that are visible for HS1(M7A), Rbcs_ HS1(M7A) and COX4_ HS1(M7A)?
- Lines 264- 277: Could the authors see if transient expression of AtFC1 would restore the phenotype. This would be the most convincing evidence.
- Line 257: EGFP:mKATE2
- Line 259: EGFP:mKATE2
- Line 149: and a high...
- In line 310: lowly-invasive would be more correct than non-invasive. Genetically encoded fluorescent sensors should always be considered as invasive.
- Sensor HS1 and its lower affinity derivate
- Lines 341-342: "The experiments using hemin supply were ambiguous and non-reproducible". Please explain further and provide possible alternative explanations.
- Lines 349-350: genetic lesion is unclear. Please reformulate.
- Lines 365: "it cannot be ruled out that overexpression of HS1 ... could have regulatory effect". Could you play with different expression levels? It could be the best evidence.
- Lines 372-373: "we conclude that the binding of free heme by HS1 does not affect the cellular role of free heme". I think it is over-concluded. I would argue further or delete.
- Lines 401-402: HS1 strategy... replace with HS1 sensor
- Lines 434-443: Could the author mention clearly which promoter has been used.
- Fig. S1-S5: The use of ratiometric data enables to get rid off potential effects from variable expression level inherent to sensor expression in vivo. Yet, it is important to provide a view of those differences between the different conditions/constructs. Could the authors put the mKate2 levels in supplementary as proxy for expression levels of the different conditions/constructs?

Reviewer #2 (Comments for the Author):

Heme is produced in plants via a plastid-localized metabolic pathway and distributed to all cellular compartments. The sensitive and effective method for spatial measuring free heme in plants is of importance. In this study, the authors applied the HS1 heme sensor and its variant HS1(M7A) to determine the relative amounts of free heme in four different subcellular compartments (cytoplasm, chloroplast, mitochondria, and nucleus) of plants. They firstly constructed HS1 and M7A variants with different

localization and quantified the relative fluorescent signals in Nicotiana benthamiana. They measured the EGFP/mKATE2 ratios in different compartments of transiently transformed N. benthamiana leaves by feeding ALA as well as incubating gabaculine. Next, they generated stable transgenic plants in Arabidopsis (expressing M7A) and N. tabacum (expressing HS1) and quantified free heme in the compartments. They also determined fluorescence ratio in N. tabacum and AtFC1-OE plants transiently expressing HS1. The authors claim that this approach makes it possible to trace the distribution and dynamics of free heme and provides relevant information about its mobilization.
Overall this is an interesting study, however, it only provides a tool without any further detailed mechanism. Here are some comments.

Major comments:
1. What's the promoter used for constructing various plasmids in figure 1A? 35S strong promoter? please clearly indicated in the text. I am wondering why the protein expression levels differ so dramatically among these constructs in Figure 1B, Figure 4A, and Figure 5A. Why does RbcS-HA1/M7A have great protein accumulation? Please explain.
2. The authors generated stable transgenic plants expressing HS1(M7A) in Arabidopsis (Figure 4) and expressing wild-type HS1 in N. tabacum (Figure 5). As stated in the first comment, the protein levels vary among different constructs. Therefore, multiple transgenic lines (at least two independent lines) for each construct in Figures 4 and 5 would be necessary to confirm the consistence of their results and conclusions. Although they showed 4 independent lines of RbcS-M7A, which showed different expression levels of M7A, then how about the other constructs?
3. The authors compared EGFP: mKATE2 ratios in wild type tobacco and transgenic plants overexpressing recombinant Arabidopsis ferrochelatase1 (AtFC1) and found that the cytosol EGFP: mKATE2 ratio decreased in FC1-OE whereas chloroplast ratio increased in FC1-OE (Figure 6). I am wondering what's the phenotype of the FC1-OE transgenic plants? Can it be explained by the EGFP: mKATE2 ratio difference?

Minor comments:
In all related figures, the number of biological repeats should be clearly provided in the figure legends.
Figure 1A, the diagram is not well presented. What's the exact length (bp) of the heme sensor? Where is the position of transit peptide? Is 247bp the right length for RbcS TP? Move heme-SV40 little bit to the right.
Figure 1D, statistical analysis might be required. Figure 1C, the size of words is not consistent.
Please indicate the method of statistical analysis in all related figures.
The scale bars were not labeled consistently.

Reviewer 1 is requesting phenotypic data of stable transformants and Reviewer 2 for FC1-OE lines. Reviewer 2 is also asking for data from multiple transgenic lines for each construct shown in Figs. 4 and 5, as done for the different Rbcs_HS1(M7A) lines (Arabidopsis or tobacco?) in Supplemental Fig. S3. Further, please provide the information on statistical tests.

Please note that any resubmission will be treated as a new entry to the system and is likely to go out for review.

If you do have any queries or concerns, you are welcome to contact me in the first instance.

Thank you again for allowing Plant Physiology to review your work. We look forward to hearing from you soon.

Sincerely,

Plant Physiology

Our Response:

We are pleased with the very positive reactions of both reviewers, who emphasize the originality of the work, speak of the high relevance of the work and highlight the lack of "overall ambiguity in the interpretations". Based on the request of reviewer 1, we provide the images of transgenic lines of each generated stable transformation of the two heme sensor variants in transgenic Arabidopsis and tobacco plants. Furthermore, the statistical data for the experiments are now added as well as immunoassays of the accumulation of the transgenic heme sensor proteins in the transformants of Arabidopsis and tobacco. We hope that these amendments will satisfy editor and reviewers.

-- Reviewer comments:

Reviewer #1 (Comments for the Author):

The authors of this manuscript describe elegantly the use of genetically encoded HS1 and HS1 (M7A) heme sensors for monitoring the distribution of free hemes in different plant cellular compartments of Arabidopsis and tobacco plants. As a proof-of-concept they used a smart strategy which combined (i) biochemical and (ii) genetic approaches to manipulate heme content by altering heme metabolism. This opens interesting new possibilities in the field of plant biology for deciphering key mechanisms in heme metabolisms, subcellular allocation, (re)distribution, dynamics, trafficking and heme-dependent signaling. Those yet numerous open questions are of high importance. Indeed, a tightly controlled labile "free" heme content is necessary for fast mobilization for heme-dependent processes while its over-accumulation should be prevented due to its toxicity potential. Despite its importance, the mechanisms or the factors involved in the control of heme distribution remains to date largely unknown.

The use of genetically encoded sensors appears to be a unique strategy in comparison to disruptive enzyme-based assays. It allows high spatial resolution with the targeting of sensors to subcellular compartment and high temporal resolution to quantify steady-state heme levels in situ which would enable accurate visualization of distribution, mobilization and trafficking of heme. This approach is particularly important as no relevant method is yet available for quantifying heme content

distribution at subcellular level and heme trafficking from the site of synthesis into the cytoplasm and other subcellular compartment.

Here, the authors adapt the sensors HS1 and HS1(M7A) from Hanna et al. 2016 for its use in plants. They have designed different constructs for their targeting to four subcellular compartments in plants (i.e., cytoplasm, plastids, mitochondria, chloroplast and nucleus). They have first hypothesized that the use of sensors such as HS1 and HS1(M7A) could enable to monitor heme content with high spatial and temporal resolutions in planta and then, have demonstrated experimentally the relevance of such an approach by using genetic, biochemical, and environmental perturbations that intend to manipulate heme contents. Their analyses demonstrate that the use of stably and transiently transformed Arabidopsis and tobacco plants expressing HS1 and HS1(M7A) enables to quantify the relative amounts of freely accessible intracellular heme and detect dynamic changes in its subcellular content. They have shown that associated affinity and expression of HS1 and HS1(M7A) are consistent with a proper function of the sensor in Arabidopsis and tobacco plants.

The importance of such a methodology is high in regards of the importance of heme-dependent proteins. Its relevance for a broad range of readers from the plant biology field is high. Especially, as few quantitative analyses have been undertaken for the relative amounts of these proteins in different subcellular compartment. A reliable method is necessary for assessing heme bioavailability expected to be tightly regulated temporally and spatially depending on the demands based on its synthesis and allocation for heme-dependent processes. The interest, originality is high while the work is highly focused. The text is clear, easy to read and presented with logic. The conception of the study as its design is relevant.

Finally, the authors provide evidences, arguments and data that support overall the conclusions while it is necessary to provide data supporting the fact that sensor expression do not affect plant developments and growth in stably transformed lines. Ine general, the conclusions respond very well to the main question. There is overall no ambiguity in the interpretations. The study will support effort aiming to decipher distribution, allocation and trafficking of free hemes in plants.

Major problem:

- In line 225, the authors stated that stably transformed Arabidopsis and tobacco lines did not show any aberrations of developments, growth rate, green pigmentation or leaf size. Sensors are considered at best lowly invasive... It is necessary to provide enough evidences/data that there is no significant alteration in plant developments and growth in derivate transgenic lines to validate the relevance of their use in vivo. Please provide data for it.

Our response: Yes, it has been done. We grew the transgenic lines again together with wild-type seedlings. In the supplemental figures 6-7,it becomes evident that neither the HS1-/HS1M7A-expressing Arabidopsis and tobacco lines in all four different subcellular compartments phenotypically differ from wild-type seedlings. We performed additional immune analyses to demonstrate the accumulation of the heme sensor variants in the different cellular compartments of always two different transgenic lines. The content of the heme senor variants did not differ in the representative lines of each transformation series Supplemental Figure 8.

Minor problems:

- HS1 and HS1(M7A) are named high and medium/lower affinity sensors by the authors. It would be good to remind their affinity somewhere in the text as high or medium/lower affinity is rather subjective.

Our response: We feel that the first version of our manuscript already contain several repeats of the requested information in the introduction and the result section (here: 1st paragraph, then 1st paragraph of the chapter "Inhibition of ALA synthesis reduces levels of subcellular free heme", first paragraph of the chapter "Transiently expressed heme sensors can be used to estimate the relative amount of free heme", and the legend of Figure 1).

- Line 7: ORCID.

Our response: The Orchid number was added when it is available

- Fig.1: The immunoblot shows a very thin signal for SV40_HS1 and SV40_HS1(M7A), please could the authors comment on this? At the opposite, Rbcs_HS1 and Rbcs_HS1(M7A) show both relatively high signals with, apparently, multiple bands. Could the authors comment on this?

Our response: We can agree with this view. It might correlate with difference in the expression intensity and/or the accumulation of the HS1 variants in the two different compartments.

- Fig.1-2: The use of ratiometric data enables to get rid off potential effects from variable expression level inherent to sensor expression in vivo. Yet, it is important to provide a view of those differences between the different conditions/constructs. Could the authors put the mKate2 levels in supplementary as proxy for expression levels of the different conditions/constructs?

Our response: If we have understood the reviewer correctly, then we can only re-emphasize that the two fluorescent proteins are co-expressed in a fusion protein and do not accumulate separately as two proteins. Evidence of the expression of the heme sensor variants is provided in figures of the manuscript and in the Supplemental Figures (new: Supplemental Figure 8).

- Fig. 1: "The EGFP: mKATE2 obtained from the GFP and mKATE2 channels was calculated from more than three transformed representative cells of different transformants." Please provide the exact numbers.

Our response: Five to eight transformed cells were used for the calculation. The information was also added in the material and method section and modified in the Figure 1 legend.

- Line 131: ALA should be defined at first mention.

- Fig. 2B: Y-axis: correct bold bracket symbols.

- Line 147: EGFP:mKATE2

our response: The three points were corrected

- In line 156: the authors claimed that it is assumed that a rise in the heme pool in the cytoplasm is a results of altered heme metabolism in plastids. I think it is over-concluded. Please argue further or delete this statement.

Our response: With reference to the sentence mentioned by the reviewer, we have amended it to achieve a more moderate wording.

- In Line 159: I am not convinced by this statement. Please argue further or delete this statement.

Our response: In this manuscript, we report the observations of a sufficient number of replicates and are currently convinced of the accuracy of our observations and thus also of this statement.

- Fig. 3B: Y-axis: correct bold bracket symbols.

Our response: Corrected

- Fig. 4A: Could the authors comment on the multiple bands that are visible for HS1(M7A), Rbcs_ HS1(M7A) and COX4_ HS1(M7A)?

Our response: We can only offer suggestions. The protein pattern is reproducible and might be due from protein degradation. We have asked Dr. Reddy also to this issue, who observed several bands in his heme-sensor expressing organisms. But he also could not offer any explanation.

- Lines 264- 277: Could the authors see if transient expression of AtFC1 would restore the phenotype. This would be the most convincing evidence.

Our response: We are not entirely sure, if we understood the comment of the reviewer correctly. Does the reviewer wish to perform a transient transformation in N. benthamiana with an AtFC1 transgene and subsequent transformation of the HS1 genes (or did the comment include the idea to use an inducible AtFC1 transgene?). We believe that these suggestions provide no further explanation, as previous FC1-overproducing lines produced phenotypes (Fan et al., 2019; see also Supplemental figure 9), which were comprehensively explained by deregulated or altered heme distribution between subcellular compartments and a likely accumulation of heme in the cytoplasm at the expense of the plastids. The phenotype of the AtFC1-expressing lines are explained by a deregulation of the heme distribution between plastids and the extraplastidal cellular compartments, and not by gene silencing. We hope that our response reflects the intention of the reviewer's comments.

- Line 257: EGFP:mKATE2

- Line 259: EGFP:mKATE2

- Line 149: and a high...

Our response : it was always corrected

- In line 310: lowly-invasive would be more correct than non-invasive. Genetically encoded fluorescent sensors should always be considered as invasive.

Our response: we modified the sentence according to the request of the reviewer.

- Sensor HS1 and its lower affinity derivate

Our response: We can accept this comment and changed the text according to the request

- Lines 341-342: "The experiments using hemin supply were ambiguous and non-reproducible". Please explain further and provide possible alternative explanations.

Our response: We regret to say that we cannot think of any other possible alternative, but we also confess that we did not pursue the trial with heme any further, as the addition of ALA caused the desired and expected effect. We have made similar experience of insufficient heme up-take in photoautotrophic Arabidopsis suspension cultures (Hampp C, Richter A, Osorio S, Zellnig G, Sinha AK, Jammer A, Fernie AR, Grimm B, Roitsch T. Establishment of a photoautotrophic cell suspension culture of Arabidopsis thaliana for photosynthetic, metabolic, and signaling studies. Mol Plant. 2012 Mar;5(2):524-7. doi: 10.1093/mp/sss018.). Heme was not taken up and/or accumulated inside the cells.

- Lines 349-350: genetic lesion is unclear. Please reformulate.

Our response: we rephrased the sentence

- Lines 365: "it cannot be ruled out that overexpression of HS1 ... could have regulatory effect". *Could you play with different expression levels? It could be the best evidence.*

Our response: We regret that we may have misled or misdirected the reviewer with this sentence. That was not our intention. Based on our intensive studies, we currently exclude the possible concern that additional heme binding of the transgenic heme sensor could disturb the heme metabolism. Based on the phenotype of all transgenic lines expressing the heme sensor variants in all four different subcellular compartments (Supplemental Figure 6 and 7), we did not observe any modification in plant growth, pigmentation, content of non-covalently bound heme (the total amount of type B-heme).

- Lines 372-373: "we conclude that the binding of free heme by HS1 does not affect the cellular role of free heme". I think it is over-concluded. I would argue further or delete.

Our response: We accept this comment and have amended and toned down the statement in line with the reviewer's wishes.

- Lines 401-402: HS1 strategy... replace with HS1 sensor

Our response: we change the word.

- Lines 434-443: Could the author mention clearly which promoter has been used.

Our response: …controlled by the35S-CaMV promoter. The information was added to the first chapter of M+M.

- Fig. S1-S5: The use of ratiometric data enables to get rid off potential effects from variable expression level inherent to sensor expression in vivo. Yet, it is important to provide a view of those differences between the different conditions/constructs. Could the authors put the mKate2 levels in supplementary as proxy for expression levels of the different conditions/constructs?

Our response : see above

Reviewer #2 (Comments for the Author):

Heme is produced in plants via a plastid-localized metabolic pathway and distributed to all cellular compartments. The sensitive and effective method for spatial measuring free heme in plants is of importance. In this study, the authors applied the HS1 heme sensor and its variant HS1(M7A) to determine the relative amounts of free heme in four different subcellular compartments (cytoplasm, chloroplast, mitochondria, and nucleus) of plants. They firstly constructed HS1 and M7A variants with different localization and quantified the relative fluorescent signals in Nicotiana benthamiana. They measured the EGFP/mKATE2 ratios in different compartments of transiently transformed N. benthamiana leaves by feeding ALA as well as incubating gabaculine. Next, they generated stable transgenic plants in Arabidopsis (expressing M7A) and N. tabacum (expressing HS1) and quantified free heme in the compartments. They also determined fluorescence ratio in N. tabacum and AtFC1-OE plants transiently expressing HS1. The authors claim that this approach makes it possible to trace the distribution and dynamics of free heme and provides relevant information about its mobilization.

Overall this is an interesting study, however, it only provides a tool without any further detailed mechanism. Here are some comments.

Our response: Of course. It is our intention to show that the generation of this heme sensor by Dr. Reddy and his group can also find application in plant cells. The mechanism of heme pool

quantification has been discussed by Dr. Reddy in original and review papers and we hope that we have sufficiently acknowledged and cited his important work. Thus, in continuation we intend to use and introduce the heme sensor. We want to demonstrate the feasibility for other researchers in the plant sector, as the reviewers have repeatedly pointed out.

Major comments:

1. What's the promoter used for constructing various plasmids in figure 1A? 35S strong promoter? please clearly indicated in the text. I am wondering why the protein expression levels differ so dramatically among these constructs in Figure 1B, Figure 4A, and Figure 5A. Why does RbcS-HA1/M7A have great protein accumulation? Please explain.

Our response: We revised the manuscript and added the information on the 35S promoter. As seen in the immunoanalysis of the accumulating heme sensor variants in the four different subcellular compartments (Supplemental Figure 8); in general, the expression of the heme sensor variants is always quite similar in the same cellular compartment of the representative lines. As we pointed out in the manuscript, the intensity of the heme sensor variants in each of the compartments likely reflects the volume of each compartment in the cells.. In green plant cells, it is assessed that chloroplast volume accounts for 40% of the total cell volume, mitochondria volume accounts for only 3-5%, the nucleus volume for even less.

Regarding the presentation of the difference in accumulation of the amounts of RBSC-M7A in four presented transgenic lines, the immunoblot should demonstrate the potential variation in protein accumulation and the similarity of the fluorescence ratio of EGFP to mKATE2. Ten transgenic lines were analyzed. Eight out of ten lines showed the higher accumulating HS1(M7A) content, while only two contain less protein. Two higher HS1 variant accumulating lines were compared with the two lines having the lower HS1(M7A) content.

2. The authors generated stable transgenic plants expressing HS1(M7A) in Arabidopsis (Figure 4) and expressing wild-type HS1 in N. tabacum (Figure 5). As stated in the first comment, the protein levels vary among different constructs. Therefore, multiple transgenic lines (at least two independent lines) for each construct in Figures 4 and 5 would be necessary to confirm the consistence of their results and conclusions. Although they showed 4 independent lines of RbcS-M7A, which showed different expression levels of M7A, then how about the other constructs?

Our response: We agree on this comment and present several lines for each transformation in the supplemental figures 6 and 7. Immune analyses confirm the expression of the heme sensor variants in always two lines for all different transgenic constructs directing the heme sensor variants to the different subcellular compartments in Arabidopsis and tobacco (Supplemental Figure 8).

3. The authors compared EGFP: mKATE2 ratios in wild type tobacco and transgenic plants overexpressing recombinant Arabidopsis ferrochelatase1 (AtFC1) and found that the cytosol EGFP: mKATE2 ratio decreased in FC1-OE whereas chloroplast ratio increased in FC1-OE (Figure 6). I am wondering what's the phenotype of the FC1-OE transgenic plants? Can it be explained by the EGFP: mKATE2 ratio difference?

Our response: we confirm the observations (see also Supplemental Figure 9) which have been already discussed in Fan et al. 2019, and Hedtke et al., 2023. The corresponding descriptions are provided in the last paragraph of the results section and in the 2nd paragraph of the discussion chapter entitled "The dynamics of heme metabolism determine the availability of heme for cellular processes."

Minor comments:

In all related figures, the number of biological repeats should be clearly provided in the figure legends.

Our response: the information is provided in the figure legends and in Materials and Methods

Figure 1A, the diagram is not well presented. What's the exact length (bp) of the heme sensor? Where is the position of transit peptide? Is 247bp the right length for RbcS TP? Move heme-SV40 little bit to the right.

Our response: Figure 1A was revised according to the requests of the reviewers.

Figure 1D, statistical analysis might be required. Figure 1C, the size of words is not consistent.

Our response: Information about the statistical analysis was always provided in all legends where requested. The size of the fonts in the images has been adjusted so that the words are uniform in size and legible.

Please indicate the method of statistical analysis in all related figures.

Our response: the revision of the manuscript contains the method of the statistical analysis in the legends where necessary and the Materials and Methods.

The scale bars were not labeled consistently.

Our response: the revision of the manuscript contains consistently labelled scale bars in the figures.

18-Apr-2024
Prof. Bernhard Grimm
Humboldt-Universität zu Berlin
Berlin 10115
Germany

MSID: PP2024-RA-00454D
MS TITLE: Application of a Genetically Encoded Fluorescent Heme Sensor to the Detection of Free Heme in Plants

Dear Dr. Bernhard Grimm:

I have read your response letter and the revised manuscript and I am pleased to inform you that your revised manuscript "Application of a Genetically Encoded Fluorescent Heme Sensor to the Detection of Free Heme in Plants" has been accepted for publication in Plant Physiology. Congratulations!

There are a few minor points I 'd appreciate if you could correct in the final version.

- You wrote: "the revision of the manuscript contains the method of the statistical analysis in the legends where necessary and the Materials and Methods." While this information is now provided in figure legends, it is still missing in the Materials and Methods.

- Please indicate the color code of the bars (hemin concentrations) in Supplemental Figure S2B.

- Supplemental Figure S6 legend: "The EGFP:mKATE2 fluorescent ratio is is derived from the the numbers of pixels in the EGFP and mKATE2 channels." Please remove the second "is" and "the".

Your manuscript will be checked for consistency with journal formatting and image standards by one of our Science Editors, who will contact you shortly. They may have a number of queries and suggestions for you. Please keep in mind that they work to increase the online visibility of your article and its attraction for readers from the widest of backgrounds. You will have the opportunity to review any revisions, and I hope you will work constructively with them to get the most out of your research. You will also have the opportunity to review all changes when you receive the author proofs for your article.

Once validated for publication, your article will be sent to a Science Editor for a final review of your manuscript and source files. After your final files are uploaded, your article will appear in the next available issue and the online Preview version of your article will be posted after your signed Copyright and Checklist forms and final source files are received.

Please be sure to include the following in your manuscript file:
Harvard style citations
List of all author contributions and funding information at the beginning of manuscript after the titles and author list.
List all supplemental materials associated with the article, with titles, at the end of the ms, before the references and acknowledgments section.
Complete Figure Legends for all figures appearing in the article listed before the Literature Cited.

Thank you and congratulations on your Plant Physiology paper!

Sincerely,
Plant Physiology

=====
FIRST AUTHOR PROFILES
=====

We recognize the hard work that goes into being First Author of a Plant Physiology paper by publishing a "First Author Feature" for each First Author alongside your paper.

If the first author(s) would like to submit information for a feature on Facebook and Twitter, fill out the form by clicking on the link below:

Plant Physiology First Author Feature Form: https://forms.office.com/r/ZBCGZfhm8F
If you cannot access the form through the link above, please contact Rachel at rbelsky@aspb.org.

To upload an image of yourself to accompany your profile, please put your photo in the correct journal folder at the link below. You must label your image: Author First Name Last Name Article Title.

First Author Profile Image Dropbox: https://www.dropbox.com/scl/fo/xz029p7qjr2jml2rp2kmq/h?dl=0&rlkey=6fkxcaqmxx35ap152b2osy1fz

=====
IMPORTANT REMINDER: PEER REVIEW REPORTS
=====

If you opted to publish a peer review report along with your article during the original submission process, it will be prepared by the editorial staff and publicly posted with your manuscript, inside the zip file that contains any other supplemental material. As a reminder, the peer review report is a public record of all comments from editors and reviewers, as well as your prior responses, as you received them in the decision letters for each draft of your manuscript. If you agreed to publish this report and have changed your mind, or are not sure if you selected this option, please contact the editorial office as soon as possible before signing the license agreement from our publisher.
